# Multi-scale detection of hierarchical community architecture in structural and functional brain networks

**DOI:** 10.1371/journal.pone.0215520

**Published:** 2019-05-09

**Authors:** Arian Ashourvan, Qawi K. Telesford, Timothy Verstynen, Jean M. Vettel, Danielle S. Bassett

**Affiliations:** 1 Department of Bioengineering, School of Engineering & Applied Science, University of Pennsylvania, Philadelphia, PA, 19104 United States of America; 2 U.S. Army Research Laboratory, Aberdeen Proving Ground, MD 21005 United States of America; 3 Department of Psychology, Center for the Neural Basis of Cognition, Carnegie Mellon University, Pittsburgh, Pennsylvania 15213 United States of America; 4 Department of Psychological & Brain Sciences, University of California, Santa Barbara, CA, 93106 United States of America; 5 Department of Electrical & Systems Engineering, School of Engineering & Applied Science, University of Pennsylvania, Philadelphia, PA 19104 United States of America; 6 Department of Physics & Astronomy, College of Arts & Sciences, University of Pennsylvania, Philadelphia, PA 19104 United States of America; 7 Department of Neurology, Perelman School of Medicine, University of Pennsylvania, Philadelphia, PA 19104 United States of America; 8 Department of Psychiatry, Perelman School of Medicine, University of Pennsylvania, Philadelphia, PA 19104 United States of America; Indiana University, UNITED STATES

## Abstract

Community detection algorithms have been widely used to study the organization of complex networks like the brain. These techniques provide a partition of brain regions (or nodes) into clusters (or communities), where nodes within a community are densely interconnected with one another. In their simplest application, community detection algorithms are agnostic to the presence of community hierarchies: clusters embedded within clusters of other clusters. To address this limitation, we exercise a multi-scale extension of a common community detection technique, and we apply the tool to synthetic graphs and to graphs derived from human neuroimaging data, including structural and functional imaging data. Our multi-scale community detection algorithm links a graph to copies of itself across neighboring topological scales, thereby becoming sensitive to conserved community organization across levels of the hierarchy. We demonstrate that this method is sensitive to topological inhomogeneities of the graph’s hierarchy by providing a local measure of community stability and inter-scale reliability across topological scales. We compare the brain’s structural and functional network architectures, and we demonstrate that structural graphs display a more prominent hierarchical community organization than functional graphs. Finally, we build an explicitly multimodal multiplex graph that combines both structural and functional connectivity in a single model, and we identify the topological scales where resting state functional connectivity and underlying structural connectivity show similar *versus* unique hierarchical community architecture. Together, our results demonstrate the advantages of the multi-scale community detection algorithm in studying hierarchical community structure in brain graphs, and they illustrate its utility in modeling multimodal neuroimaging data.

## Introduction

Hierarchical organization is a common motif in information processing systems [1]. The local embedding of similar processing units within groups that are then iteratively combined into larger and larger subsystems [2] provides a unique solution to the problem of balancing information segregation (within a group at a single scale) and integration (between groups across multiple scales) [3]. Such an organization is observed in very large-scale computer circuits and computing architectures [4, 5], cellular communication systems [6], and social messaging systems [7]. Across these various real-world information processing systems, hierarchical organization can additionally offer robustness to damage [8, 9], and a complex and diverse repertoire of system functions [10, 11] that promote optimal and efficient information processing [12, 13] and transmission.

While these previous examples are all man-made systems, hierarchical organization is also present in natural information processing systems. A quintessential example is the brain—whether dissected in a nematode worm such as *C. elegans*, or non-invasively measured in a healthy adult human [14]. Importantly, hierarchies in these systems can occur in both time [15] and space [1], and can exist in the clustering of gene expression [16] or the groupings of neuronal cell types in lamina and columns [17]. Arguably one of the most complex types of architecture in the brain is hierarchical *network* architecture [18, 19]. Here, brain regions are represented as network nodes, and structural or functional connections are represented as network edges [20]. Both structural and functional networks in the brain are critical conduits for information flow, processing, transmission, and cognitive computations more generally [21]. Yet, despite its fundamental importance, our understanding of the hierarchical organization in the brain remains limited, in part due to the fundamental nature of complex networks: they defy visual interpretation, and instead require computational algorithms to characterize.

Algorithmic methods to identify hierarchical network architecture must overcome the challenge of identifying embedded processing units within local groups. Particularly useful candidates include community detection methods, which currently dominate the study of brain networks [22]. Community detection techniques can take on many forms [23, 24], but perhaps the most common in the context of neuroimaging data is modularity maximization [25]. In this approach, nodes are partitioned into communities such that nodes within a community are more likely to connect to one another than expected in a random network null model [26]. Importantly, the size of communities identified can be tuned by a structural resolution parameter, which titrates the relative difference between the real intra-community density and that expected in the null model [27, 28]. Therefore, sweeping across a range of resolution parameters offers glimpses into the hierarchical organization of the graph [29, 30]; however, since the communities are identified independently at each point along the sweep, a secondary algorithm is required to track or link communities across topological scales, for example based on the similarity between communities in neighboring slices.

To address this limitation, we use a multi-scale community detection algorithm recently developed in applied mathematics [31] to retrieve the underlying hierarchical organization of both artificial graphs and graphs representing human brain connectivity. We find that multi-scale community detection carefully preserves local information about the stability of sub-communities in the graph, enabling a thorough description of its hierarchical levels. Perhaps even more interestingly, we can uncover communities that remain stable across topological scales, and we can characterize their longevity and frequency. This approach offers unique advantages—such as sensitivity to community longevity—that extend more common methods that sweep across global topological scales [29, 32] with independent estimates. Finally, we present methods for statistical assessment of the identified hierarchical communities, and we further offer an approach for the estimation of a consensus partition across the hierarchy.

We exercise and apply this multi-scale approach to better understand the putative hierarchical community organization of patterns of white matter pathways (SC) estimated from diffusion spectrum imaging, and of functional connections (FC) estimated from resting state functional magnetic resonance imaging. Across 60 healthy adult individuals, we show that SC is topologically heterogeneous, displaying a varying number of stable communities across brain regions. In contrast, we show that the hierarchical organization of FC is flatter, displaying a smaller number of stable communities across scales. Building on these observations, we probe the spatial embedding of communities in each modality separately, and then we compare and contrast the two modalities in a statistical sense, while acknowledging that the generative parameters and methods of constructing these graphs are inherently different. Our work offers a roadmap for the use of multi-scale community detection in revealing hierarchical network structure in structural and functional brain graphs, and in assessing their relationships to one another. In future research, this technique could used to better understand multimodal hierarchical architectures in disease, development, and aging, and in different cognitive states.

## Methods

### Participants

Sixty participants (28 male, 32 female) were recruited locally from the Pittsburgh, Pennsylvania area as well as the U.S. Army Research Laboratory in Aberdeen, Maryland. Participants were neurologically healthy adults with no history of head trauma, neurological pathology, or psychological pathology. Participant ages ranged from 18 to 45 years old (mean age, 26.5 years). The study protocol for acquiring the human subjects data was reviewed and approved by the IRB at Carnegie Mellon University and written informed consent was obtained for all participants. As the present work uses de-identified human data from the original CMU study, the Penn IRB deemed this study exempt from the requirement for ethical review.

### MRI acquisition

All 60 participants were scanned at the Scientific Imaging and Brain Research Center at Carnegie Mellon University on a Siemens Verio 3T magnet fitted with a 32-channel head coil. An MPRAGE sequence was used to acquire a high-resolution (1 mm^3^ isotropic voxels, 176 slices) T1-weighted brain image for all participants. DSI data was acquired following fMRI sequences using a 50 min, 257-direction, twice-refocused spin-echo EPI sequence with multiple *q* values (*TR* = 11, 400*ms*, *TE* = 128*ms*, voxel size 2.4 mm^3^, field of view 231 × 231 mm, *b*-max 5000 s/mm^2^, 51 slices). Resting state fMRI (rsfMRI) data consisting of 210 T2*-weighted volumes were collected for each participant with a BOLD contrast with echo planar imaging (EPI) sequence (TR 2000 ms, TE 29 ms, voxel size 3.5 mm^3^, field of view 224 × 224 mm, flip angle 79 degrees).

Head motion is a major source of artifact in resting state fMRI data (rsfMRI). Although recently developed motion correction algorithms are far more effective than typical procedures [33–36], head motion was additionally minimized during image acquisition with a custom foam padding setup designed to minimize the variance of head motion along pitch and yaw directions. The setup also included a chin restraint that held the participant’s head to the receiving coil itself. Preliminary inspection of EPI images at the imaging center showed that the setup minimized resting head motion to 1 mm maximum deviation for most subjects. Only 2 subjects were excluded from the final analysis because they moved more than 2 voxels multiple times throughout the imaging session.

### Diffusion MRI reconstruction

DSI Studio (http://dsi-studio.labsolver.org) was used to process all DSI images using a *q*-space diffeomorphic reconstruction method [37]. A nonlinear spatial normalization approach [38] was implemented through 16 iterations to obtain the spatial mapping function of quantitative anisotropy (QA) values from individual subject diffusion space to the FMRIB 1 mm fractional anisotropy (FA) atlas template. QA is an orientation distribution function (ODF) based index that is scaled with spin density information that permits the removal of isotropic diffusion components from the ODF to filter false peaks, facilitating the resolution of fiber tracts using deterministic fiber tracking algorithms. For a detailed description and comparison of QA with standard FA techniques, see [39]. The ODFs were reconstructed to a spatial resolution of 2 mm^3^ with a diffusion sampling length ratio of 1.25. Whole-brain ODF maps of all 60 subjects were averaged together to generate a template image of the average tractography space.

### Fiber tractography and analysis

Fiber tractography was performed using an ODF-streamline version of the FACT algorithm [39] in DSI Studio, using the builds from September 23, 2013 and August 29, 2014. All fiber tractography was initiated from seed positions with random locations within the whole-brain seed mask with random initial fiber orientations. Using a step size of 1 mm, the directional estimates of fiber progression within each voxel were weighted by 80% of the incoming fiber direction and 20% of the previous fiber direction. A streamline was terminated when the QA index fell below 0.05 or had a turning angle greater than 75 degrees. We performed a region-based tractography to isolate streamlines between pairs of regional masks. All cortical masks were selected from an upsampled version of the original Automated Anatomical Labeling Atlas (AAL) [40, 41] containing 90 cortical and subcortical regions of interest but not containing cerebellar structures or the brainstem. This resampled version contains 600 regions and is created via a series of upsampling steps in which any given region is bisected perpendicular to its principal spatial axis in order to create 2 equally sized sub-regions [42]. The final atlas contained regions of an average size of 268 voxels, with a standard deviation of 35 voxels. Diffusion-based tractography has been shown to exhibit a strong medial bias [43] due to partial volume effects and poor resolution of complex fiber crossings [44]. To counter the bias away from more lateral cortical regions, tractography was generated for each cortical surface mask separately.

### Resting state fMRI preprocessing and analyses

SPM8 (Wellcome Department of Imaging Neuroscience, London) was used to preprocess all rsfMRI collected from 53 of the 60 participants with DSI data. To estimate the normalization transformation for each EPI image, the mean EPI image was first selected as a source image and weighted by its mean across all volumes. Then, an MNI-space EPI template supplied with SPM was selected as the target image for normalization. The source image smoothing kernel was set to a FWHM of 4 mm, and all other estimation options were kept at the SPM8 defaults to generate a transformation matrix that was applied to each volume of the individual source images for further analyses. The time-series was up-sampled to a 1Hz TR using a cubic-spline interpolation. Regions from the AAL600 atlas were used as seed points for the functional connectivity analysis [42]. A series of custom MATLAB functions were used to extract the voxel time series of activity for each region, and to remove estimated noise from the time series by selecting the first five principal components from the white matter and CSF masks.

### Data preprocessing

The DSI and BOLD data were used to construct *N* × *N* structural and functional networks, respectively, where *N* = 600 regions. We then studied the hierarchical community structure of these graphs using multi-scale community detection.

#### Functional network construction

Following prior work [45], we estimated the functional connectivity between all region pairs using a wavelet coherence [46]. We choose the wavelet decomposition based on its denoising properties [47] and its utility in estimating statistical similarities between long memory time series such as those observed in resting state fMRI data [48]. We observe two distinct bands of high coherence: 0.24 − 0.17*Hz* and 0.16 − 0.08*Hz*. We focus on the 0.16 − 0.08*Hz* band due to its known sensitivity to underlying neural activity [49]. Coherence amplitudes were averaged over all frequencies and time points within the selected band to construct the average band-passed coherence for each pair of regions resulting in a single *N* × *N* functional connectivity (FC) adjacency matrix per subject [50].

#### Structural network construction

The individual subject’s structural connectivity (SC) matrix represents the fiber count between all region pairs. Commonly the fiber count values are normalized by the region size, so that the values of the SC matrix reflect the *density* of the white matter streamlines constructed between two regions [51]. However, we circumvented this issue by using the AAL600 atlas [42], which was purposefully designed to contain similarly-sized regions. Due to the heavy tailed nature of the edge weight distribution (see S2 Fig) we applied a log transform to the edge weights (log(*SC* + 1)/max(log(*SC* + 1))). This transformation of the data served to increase the discriminability of low edge weights, and to increase the comparability of the structural edge weight distribution and the functional edge weight distribution.

### Community detection

Common community detection algorithms can be used to partition a graph into clusters, where nodes tend to be more tightly connected to other nodes in their same cluster than to nodes in other clusters [23, 24]. In the context of network data (or other relational data that can be represented as a network), we adopt common parlance and refer to these clusters as *communities*. A common heuristic for the identification of community structure in network data is the optimization of a quality function, which measures the relative density of the intra- *versus* inter-community edges [25, 52]. One particularly popular quality function is the *modularity* quality function [53], which can be defined as:

$$\begin{array}{c} Q=\sum_{ij}[(A_{ij}-\gamma P_{ij})]\delta (g_{i},g_{j}), \end{array}$$ (1)

where for a graph of *N* nodes, *A* is the *N* × *N* weighted adjacency matrix, the *ij*^*th*^ element of the adjacency matrix indicates the weight of the connection between node *i* and node *j*, the Kronecker delta *δ*(*g*_*i*_, *g*_*j*_) = 1 if the community assignment of node *i* and node *j* (*g*_*i*_, *g*_*j*_) are identical (*g*_*i*_ = *g*_*j*_) and zero otherwise, *γ* is the structural resolution parameter, and *P*_*ij*_ is the expected weight of the *ij*^*th*^ edge between node *i* and node *j* under a specified null model. Importantly, by maximizing this quality function, one can identify a partition of nodes into communities. However, identifying the optimal partition is NP-hard, and therefore clever heuristics such as the Louvain-like locally greedy algorithm are often used to seek near-optimal partitions [54]. To account for the degeneracy of the modularity landscape [55], the algorithm is used to optimize the modularity quality function multiple times, and results that remain consistent over those optimizations are reported [56].

The Newman-Girvan null model [57] is the most commonly used null model in modularity maximization. It can be defined as: $P_{ij}=\frac{k_{i}k_{j}}{2m}$, where *k*_*i*_ = ∑_*j*_ *A*_*ij*_ is the strength of node *i* and $m=\frac{1}{2}\sum_{ij}A_{ij}$. This null sets the expectations of an edge based on the strength of its nodes. The structural resolution parameter *γ* is commonly chosen to equal unity [58–60]. However, by using only this single value of *γ*, the investigator is only accessing a single topological scale of the network’s community structure [14, 56]. Because graphs often display hierarchical organization, it is frequently useful to explore community structure in a graph over a range of values for *γ* [14, 29, 30, 56]. When a graph has a particularly salient topological scale at which community structure is strongest, this parameter sweep can be used to identify the structural resolution parameter value at which that community structure can be identified [56]. However, for a graph that has hierarchical structure in which multiple topological scales are equally salient, this approach can fail to identify a single useful structural resolution parameter value.

### Hierarchical community detection

**Fig 1:**
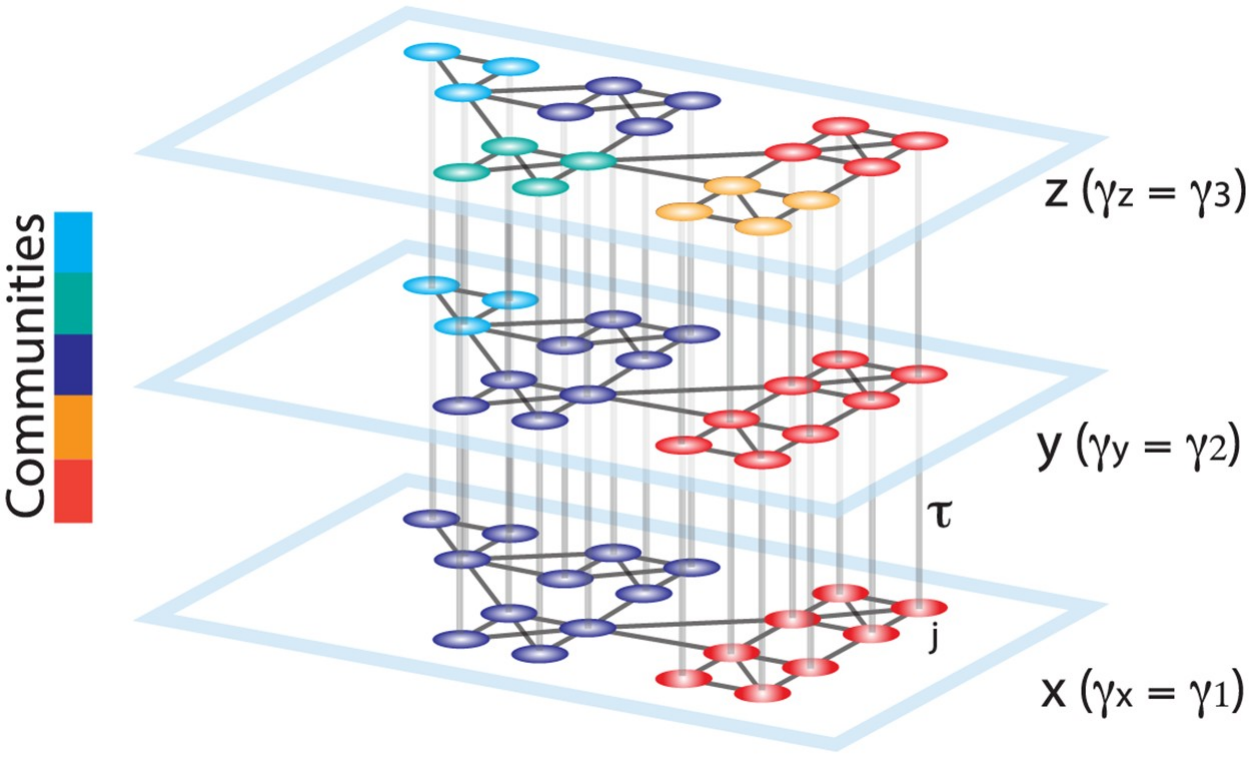
Schematic representing the construction of a multilayer network for use in multi-scale modularity maximization. Duplicates of a graph are connected in a multilayer fashion to construct a 3D graph. The smallest resolution parameter *γ* is assigned to the first layer (*x*), and it is linearly increased for the neighboring layers (*y*, *z*). The topological scale coupling parameter, *τ*, tunes the strength of the dependence of communities across layers. Since the community assignments are dependent on the adjacent layers, nodes that display high clustering over neighboring topological scales are identified as a single community spanning several scales. In this schematic, the large communities identified at initial layers progressively break into smaller sub-communities, revealing the hierarchical community organization of the graph.

To study hierarchical community structure in graphs, we suggest a method based on optimizing the modularity quality function across all neighboring topological scales simultaneously. Specifically, we create a multilayer network [31], linking duplicates of the graph at each *γ* value to the graphs at neighboring *γ* values. For a schematic representation of the proposed multilayer graph, see Fig 1. Formally, we define the multi-scale modularity quality function as:

$$\begin{array}{c} Q=\frac{1}{2\mu }\sum_{ijxy}\{(A_{ij}-\gamma_{x}P_{ij})\delta_{xy}+\delta_{ij}\tau_{jxy}\}\delta (g_{ix},g_{jy}), \end{array}$$ (2)

where the *ij*^*th*^ element of the adjacency matrix **A** indicates the weight of the connection between node *i* and node *j*, the Kronecker delta *δ*(*g*_*ix*_, *g*_*jy*_) = 1 if the community assignments of node *i* from scale *x* and node *j* from scale *y* (*g*_*ix*_, *g*_*jy*_) are identical (*g*_*ix*_ = *g*_*jy*_) and zero otherwise, *γ*_*x*_ is the structural resolution parameter at layer *x*, *P*_*ij*_ is the expected weight of the edge between node *i* and node *j*, *τ*_*jxy*_ is the *topological scale coupling parameter* which indicates the strength of the links between neighboring topological scales (as represented by layers), the total edge weight in the network is $\mu =\frac{1}{2}\sum_{jy}^{}K_{jy}$, the strength of node *j* in layer *y* is *K*_*jy*_ = *k*_*jy*_ + *c*_*jy*_, the intra-layer strength of node *j* in layer *y* is *k*_*jy*_ = ∑_*i*_ *A*_*ij*_, and the inter-layer strength of node *j* in layer *y* is *c*_*jy*_ = ∑_*x*_ *τ*_*jxy*_. Here we studied the simplified case in which *τ* is chosen identically for all nodes and all layers.

In formulating **P**, we consider a geographic null model [61], sometimes also referred to as a constant Potts model [60], where the expected value of the edge *P*_*ij*_ is uniform for all edges. This null is typically chosen to obtain a community partition built from information housed in the original adjacency matrix without discounting within-community density by the degree of the nodes that comprise the community (as is done in the Newman-Girvan null model). The geographic null is also sometimes used to circumvent the resolution limit of many other commonly used community detection methods [60]. In our context, we have a third reason to choose this null model, which is that it serves to preserve the one-to-one relationship between edge weight and the topological scales where communities are identified. This advantage enhances the interpretably of the identified topological scales, because they directly correspond to a certain range of edge weights similarly across the nodes of the graph, despite the presence of marked heterogeneity in node degree.

When using a geographic null model in modularity maximization, one can change the value of the constant null without affecting the hierarchical community structure, but instead simply shifting the range of *γ* values spanning from a partition of network nodes into *N* communities (the finest scale of the hierarchy) to a partition of network nodes into 1 community (the coarsest scale of the hierarchy). In studies that consider graphs from multiple subjects, it is critical for the purposes of accurate inference to ensure that the community structure obtained from one subject at a given *γ* value is at the same scale of the hierarchy as the community structure obtained from another subject at that same *γ* value. To ensure this comparability, we choose a constant null that aligns the topological scale of community structure across subjects for a given *γ*, and this choice can naturally differ in different data types (such as structural and functional). In the structural matrices, we noted that min(*A*_*ij*_) is relatively consistent across the subjects in our sample (being equal to 1 streamline), while mean(*A*_*ij*_) is quite different across subjects in our sample. Thus, an appropriate choice for the null constant is min(*A*_*ij*_). In functional matrices, we noted that mean(*A*_*ij*_) was more consistent across subjects than min(*A*_*ij*_) in our sample. Thus, an appropriate choice for the null constant is mean(*A*_*ij*_). Note that these choices ensure appropriate inferences across subjects for the two data types separately. Moreover, they also allow appropriate inferences across data types because relative differences in *γ* values can be interpreted identically.

The *τ* parameter tunes the dependence of communities across neighboring layers, which in our case track topological scales in the network. Intuitively, as the value of *τ* tends toward 0, the method identifies community structure that is independent across topological scales, while at high values of *τ* the method identifies a community structure that is largely shared across topological scales. Clear hierarchical structure, where small modules are nested inside larger modules, is accessible at middling values of *τ*. This intuition can be obtained directly from the structure of Eq 2, as well as from a visual inspection of numerical simulations performed over different values of *τ* (S1 Fig). Because of our interest in hierarchical architecture, we only examine the multi-scale community structure of the FC and SC graphs at a middling value of the topological scale coupling parameter (*τ* = 0.5) where the community organization of the neighboring topological scales does not exhibit complete dependence nor complete independence. Finally we note that while not the focus of this paper, it is important to note that the hierarchical community detection framework that we describe and exercise can also be extended to other multi-layer and temporal graphs. In the latter case, our framework can be used to link communities across different temporal scales; see section 1 in the appendix entitled “Spectral community detection of dynamic (multi-slice) networks” for details.

### Statistics of multi-scale communities: Stability and consensus communities

The multi-scale community detection algorithm identifies many communities that could span several topological scales, each here represented as a layer in the multilayer network. In other similar multilayer contexts, it is crucial to be able to assess the stability of the identified communities across scales [29], under the assumption that stable communities are of particular interest [62]. In our multi-scale framework, we measure the stability of individual nodes’ allegiance to their communities across scales: for node *i* the stability of its allegiance to community *’X’* is calculated as the number of *γ* values where node *i* belongs to community *’X’* divided by the total number of slices. Recall that the total number of slices is given by the number of structural resolution parameter values examined. Importantly, we independently calculated the stability values of each node’s allegiance to all communities to which it belongs, regardless of their size, across all hierarchical scales. Higher values of stability indicate that a node belongs to a single community across a greater number of layers, indicating its participation in a wider range of topological scales in the hierarchy. Importantly, this notion of allegiance across hierarchies is made possible by the multi-scale formulation, which allows a community in one scale to be associated statistically with a community at a different scale.

Importantly, a node’s stability can be calculated as a function of the value of the structural resolution parameter *γ*. For example, suppose node *i* is assigned to community *’X’* at *γ* = 1 and community *’Y’* at *γ* = 2. The stability of node *i* at the point *γ* = 1 is then equal to the number of *γ* values where node *i* belongs to community *’X’* divided by the total number of slices; by contrast, the stability of the same node *i* at the point *γ* = 2 is equal to the number of *γ* values where node *i* belongs to community *’Y’* divided by the total number of slices. Thus, in fact we can calculate a stability matrix that encodes the stability of each node at each value of the structural resolution parameter (see S3A Fig). In this matrix, highly diverse patterns of stability are indicative of topological heterogeneity in the graph. By constrast, less diverse patterns of stability are indicative of topological homogeneity in the graph.

Because we employ a heuristic to maximize the modularity quality function [54], the identified partition of the multilayer network into multi-scale communities can change at each iteration [55]. To establish a robust, representative partition across these iterations, we perform the following steps: (i) we maximize the modularity quality function many times (*n* = 100) to adequately sample the modularity landscape, (ii) for every pair of brain regions, we calculate the average probability of two nodes appearing in the same community (which we refer to as the *intra-layer community allegiance*) from the multi-scale partitions for all layers, (iii) for every brain region, we calculate the average probability of it appearing in the same community across two neighboring layers (which we refer to as the *inter-layer community allegiance*) from the multi-scale partitions for all neighboring layers, (iv) we identify the nodes (and layers) with reliable inter- and intra-layer community allegiance by comparing average community allegiance values with that of a null model. The average community allegiance of the null model was generated by randomly permuting community labels from step (i). Then, (v) following [56], we create a consensus multi-layer graph where the weights of the intra-layer and inter-layer edges correspond to the average community allegiance of the edges that were found to be significantly different from the null model. All of the non-significant edges were removed from the multi-layer graph. Finally, (vi) the consensus partition is identified from the multi-layer consensus graph using the multi-layer community detection algorithm [31] with a resolution parameter value of *γ* = 1 and an inter-layer coupling parameter value of *ω* = 1.

## Results

### Hierarchical community organization of synthetic graphs

**Fig 2:**
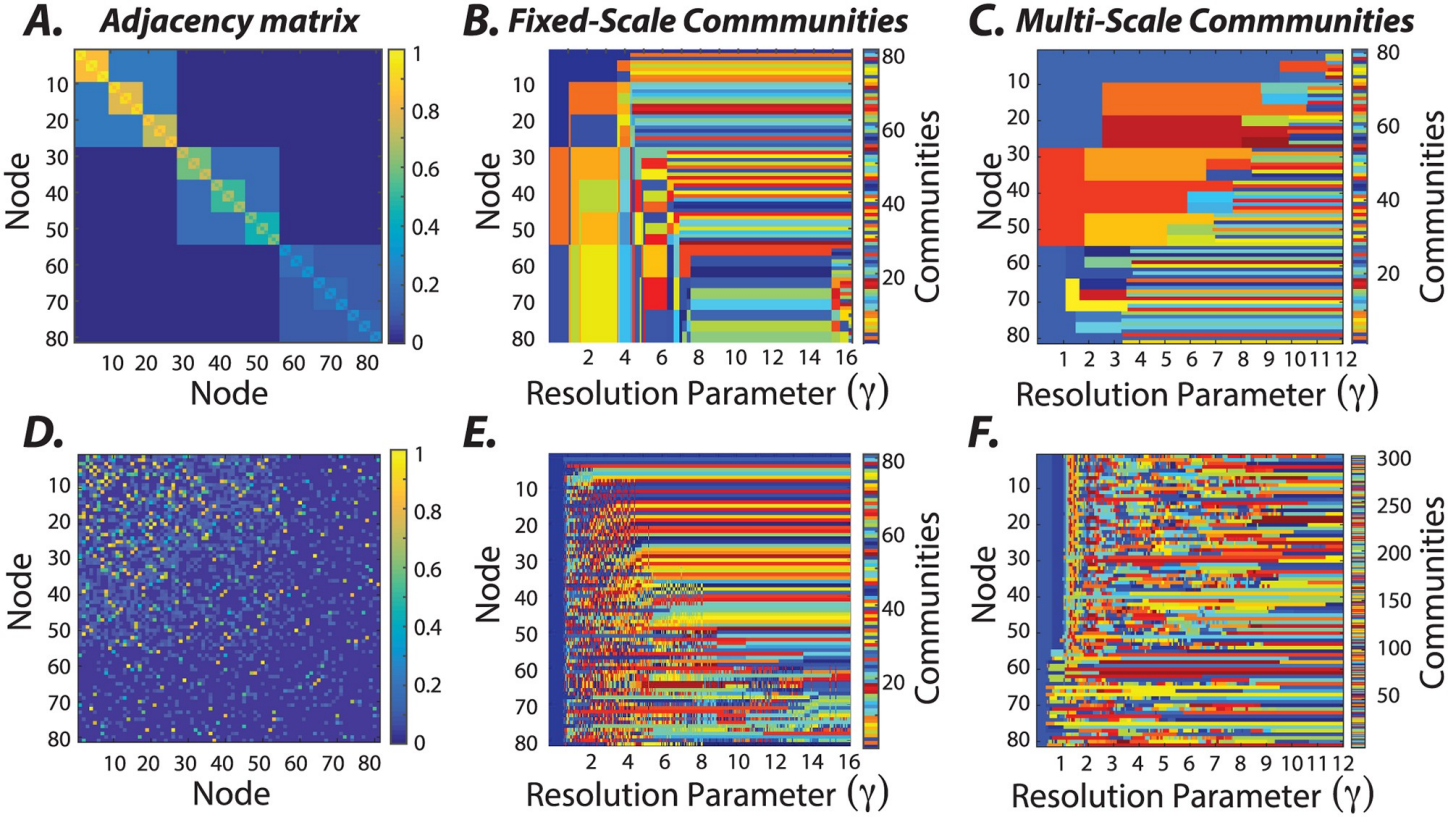
Uncovering hierarchical community structure in a synthetic graph. *(A)* Graphs can display heterogeneity in hierarchical community structure. To gain intuition regarding the utility of our method for characterizing these sorts of graphs, we design a synthetic graph such that each node is part of a small cluster composed of 3 nodes, a medium sized cluster composed of 9 nodes, and a large cluster composed of 27 nodes. Topological heterogeneity is introduced by adding gradients in the values of edge weights such that not all clusters of a given size have the same average weight. *(B)* We seek to uncover the hierarchical structure in this synthetic graph. First, we use the more traditional approach of maximizing a single-layer modularity quality function [53] with the Newman-Girvan null model [57] using a Louvain-like locally greedy algorithm [54]. We sweep the resolution parameter between 1 and 16, and identify communities independently at each *γ* value. The limitation of this approach is that there is no guarantee that communities at one resolution correspond to communities at another resolution. *(C)* To overcome this limitation, we next seek to uncover the hierarchical structure in this synthetic graph using a multi-scale approach built on multi-scale community detection [31]. We find that the hierarchical community detection uncovers the true underlying community organization as we vary the value of the structural resolution parameter (*γ* ∈ [0, 12], *τ* = 0.05, an inter-layer *γ* increment of 0.05, and 241 layers). Moreover, the *γ* value at which a community is detected tracks the mean edge weight of the community; stronger communities are identified at larger *γ* values, and weaker communities are identified at smaller *γ* values. *(D)* For comparison, we created a null graph without any clear hierarchical community structure by randomly shuffling the edges of the graph in panel *(A)*, while preserving the weight, degree, and strength distributions (see [63] for details). Panels *(E)* and *(F)* show the communities detected in this null graph when using the parameter sweep method and the multi-scale community detection method, respectively. Note that the lack of hierarchy in the null graph is similarly echoed in the multi-scale community structure displayed in panel *(F)*, marked by predominantly small and unstable communities across topological scales.

To illustrate the method, we begin with a synthetic hierarchical graph that is constructed so as to contain clear community structure across a range of topological scales (Fig 2A). Specifically, the graph displays identifiable community structure across four topological scales, with nested clusters of 3, 9, and 27 nodes. Heterogeneity is introduced by adding gradients in the values of edge weights such that not all clusters of a given size have the same average weight. To uncover the hierarchical community structure in this synthetic graph, we first applied an existing approach: a maximization of a single-layer modularity quality function [53] with the Newman-Girvan null model [57] using a Louvain-like locally greedy algorithm [54]. Across different values of the structural resolution parameter (*γ*), we observe that the communities identified appear to change frequently, with different communities being present at different values of *γ* (Fig 2B). Prior work suggests that a reasonable method to choose a useful value for the structural resolution parameter is to identify a range of *γ* over which the community structure does not change appreciably [29, 56]. Applying that approach to these data, one might identify 9 < *γ* < 15 as a range of *γ* values over which the community structure is relatively stable (Fig 2B). Yet, the community structure present in this range of *γ* values alone does not reflect the planted hierarchical organization and the topological inhomogeneities across nodes, as seen in Fig 2A. It is worth noting that the community labels across different *γ* values (Fig 2B) are unrelated as the communities are identified independently for each *γ*. This mismatch is known as the correspondence problem, and is typically addressed by using an additional algorithm to establish the relationship between the community labels across topological scales.

To overcome the correspondence problem, we apply a multi-scale community detection method using the hierarchical algorithm described in the Methods section. We observe that the community structure displays branching across layers (or values of *γ*; Fig 2C), meaning that a community in one layer can branch into two or more subcommunities in the next layer. Tracking the changes in a node’s community allegiance as a result of branching into subcommunities gives us information about the local topology of the synthetic graph. These results suggest that the multi-scale community detection technique can accurately uncover planted hierarchical communities in synthetic graphs. To gain further intuition regarding the performance of the method, we also apply the approach to three other synthetic graphs with differing architectures (S4 Fig). Again, we observe that the community structure displays branching across layers and that the number of branches is indicative of the number of local hierarchical scales in the synthetic graph. Moreover, across all synthetic graphs, we can observe that communities with higher-valued edge weights appear at higher values of *γ* than communities with lower-valued edge weights. Lastly, to demonstrate the utility of the multi-scale community detection method in probing the community organization of graphs, we consider its application to a null graph that does not have any clear hierarchical community structure. We constructed the null graph by randomly permuting the location of edges from the graph in Fig 2A while preserving weight, degree, and strength distributions (for more details see [63]). We show the resultant null graph as well as the communities identified from it using the *γ* parameter sweep method and the multi-scale community detection method in Fig 2D–2F, respectively. Note that the multi-scale method identifies a large number of small and unstable communities, without any clear hierarchical organization, consistent with the underlying ground truth. Together, these examples highlight the utility of the multi-scale community detection method for revealing hierarchically organized communities when they exist.

### Hierarchical community organization of white matter structure in the human brain

**Fig 3:**
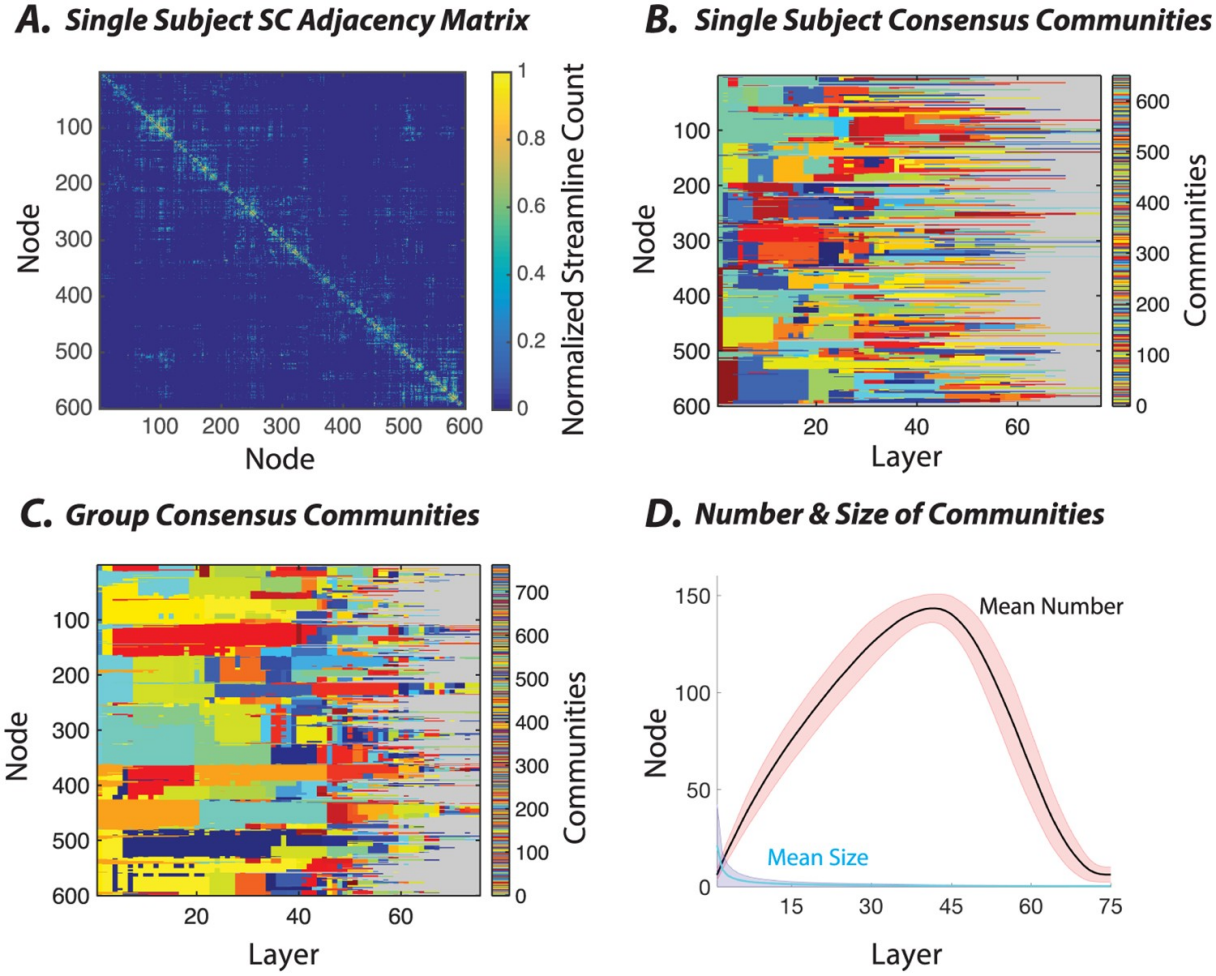
Application of multi-scale community detection to subject-level and group-level structural brain networks. *(A)* An example structural connectivity matrix from one subject, in which each element linking a pair of brain regions represents the number of streamlines reconstructed between those two areas. To better visualize its structure, we apply a log transformation (log(*SC* + 1)/max(log(*SC* + 1))). *(B)* The consensus partition representing the multi-scale community structure for the matrix in panel *(A)*. To enhance the visual detection of communities, we have represented all singleton communities with the same gray color. *(C)* The group-level consensus partition representing the multi-scale community structure for the structural matrix, which is defined as the consensus over all participants’ partitions. Here, again, to enhance visual clarity, we color the singleton communities in the same gray color. *(D)* The average number (out of 600 total nodes) as well as the average size (expressed as the percentage of total nodes) of the non-singleton communities calculated across layers, which in our case represent *γ* increments. In these analyses, we used *γ* ∈ [0.0133, 1], an inter-layer *γ* increment of 0.0133, and 75 layers.

For a given subject, the structural connectivity (SC) matrix generated from the fiber count between 600 brain parcels is sparse, with an average streamline count of 3.57 and a standard deviation of 37.83 (see Methods). Intuitively, this sparsity indicates that relatively few brain regions share direct fiber connections with one another. Moreover, the distribution of edge weights is heavy tailed (see S2 Fig), ranging from only a few streamlines per node pair to several thousand per node pair. To better visualize the architecture of the SC matrix, we apply a log transform (log(*SC* + 1)/max(log(*SC* + 1))) (see Fig 3A). Then, we use the multi-scale community detection method to determine whether the SC matrix displays hierarchical community structure, and if it does, to characterize that structure both qualitatively and quantitatively.

In single subjects, we observe that the SC graphs display hierarchical organization where communities branch into smaller sub-communities across a range of topological scales (Fig 3B). These characteristics of single-subject SC graphs are recapitulated at the group level. By performing consensus clustering, we can estimate a hierarchical decomposition that is most characteristic of all subjects within the group. We observe a similar hierarchical community structure, indicating a high degree of similarity (and low variance) across subjects (Fig 3C). In the S5 and S6 Figs, we show that these group-level communities tend to be composed of spatially proximal, or connected, regions indicating that topological clustering is nontrivially related to spatial location. An interesting exception to this general trend is the existence of a few spatially distributed communities located in the fronto-striatal circuitry that bridge frontal cortex and the striatum.

The hierarchical community organization in the SC graphs can be described by characteristic curves of community number and size as a function of resolution. Specifically, as the value of the structural resolution parameter increases, we observe a rapid increase in the number of non-singleton communities (maximum average number of SC communities = 143.20 ± 7.3 std) and an analogous drop in the average community size (Fig 3D). As the value of *γ* increases farther, the number of communities branching into singletons increases, and thus the number of non-singleton communities decreases. Together, these trends indicate that communities begin to branch at low *γ* values, and continue to branch as *γ* increases (full width at half max = 42.49 ± 2.9, Fig 3D). Far from haphazard, this global branching process is highly structured, with a large number of nodes maintaining their allegiance to hierarchical communities over a long range of *γ* before branching (see Fig 3B).

### Hierarchical community organization of functional connections in the human brain

The functional connectivity (FC) values were calculated based on the average wavelet coherence between regional time series, and unlike the SC matrix values, they exhibit a normal distribution with an average value of ≈ 0.49 and relatively small standard deviation (≈ 0.04) (S2C and S2D Fig). Intuitively, this narrow range of edge weights means that FC graphs will display hierarchical organization over a smaller range of *γ* values. It is important to note that when considering any specific network type, the critical goal is to identify a range of *γ* parameters that will span the full range of community structure present in the data. Thus, one wishes to first identify the *γ* parameter that leads to *N* communities, where *N* is the number of nodes. This is the finest level of community structure present in the data. Next, one wishes to identify the *γ* parameter that leads to 1 community. This is the coarsest level of community structure present in the data. Sweeping between these two *γ* values allows a comprehensive characterization of all levels of community structure present in the data. Accordingly, to best sample the FC hierarchical structure, we therefore assessed community organization over a smaller range of *γ* values, with *γ* ∈ [0.95, 1.7] for the FC matrices as opposed to the *γ* ∈ [0.0133, 1] used for the SC matrices. Importantly, these ranges were chosen pragmatically so as to map out the entire curve from partitions containing a single community (lowest value in the *γ* range), to partitions containing only singletons (highest value in the *γ* range).

**Fig 4:**
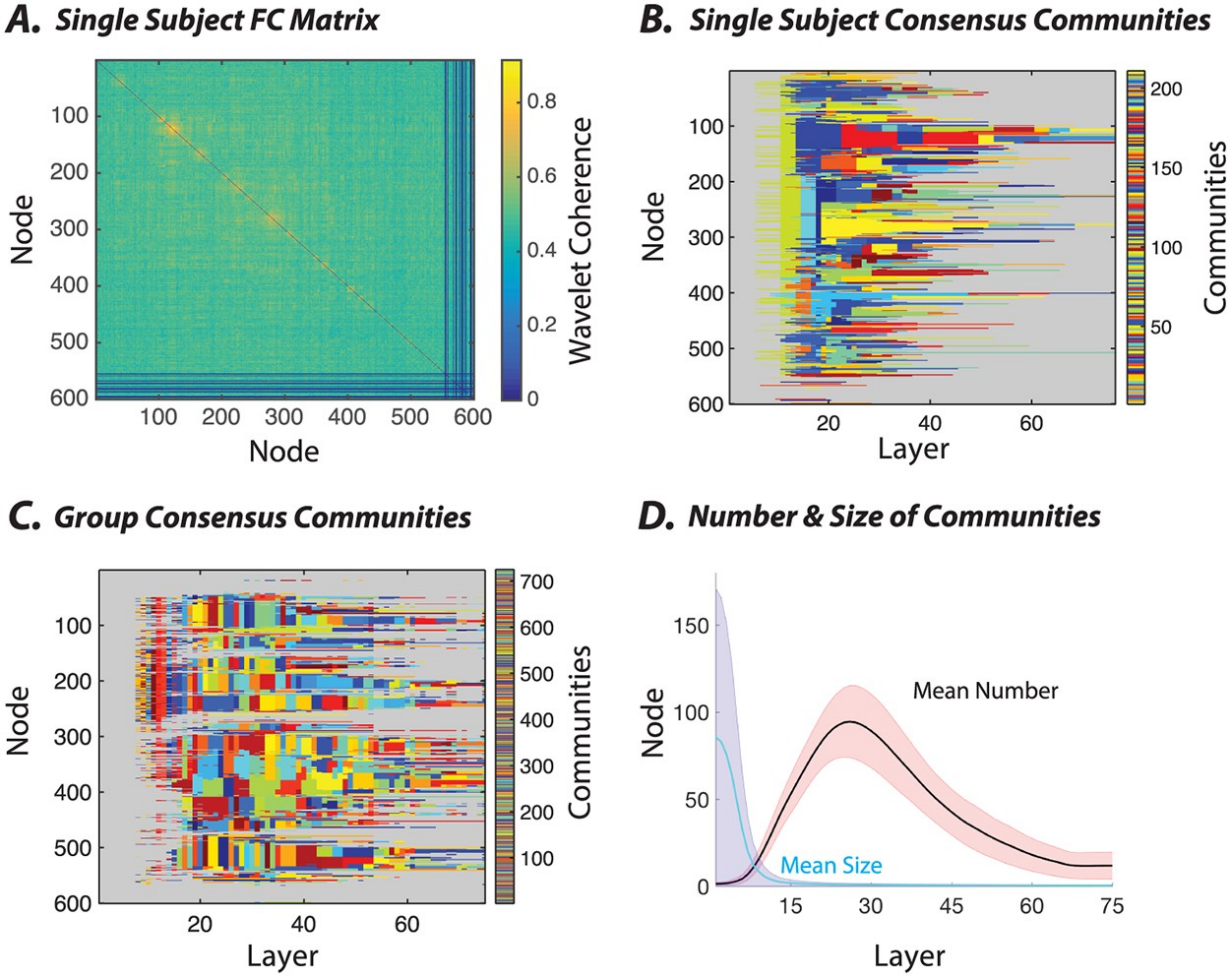
Application of multi-scale community detection to subject-level and group-level resting state functional brain networks. *(A)* An example rsfMRI connectivity matrix from one subject, in which each element linking a pair of brain regions represents the pairwise wavelet coherence between regional time series. *(B)* The consensus partition representing the multi-scale community structure for the matrix in panel *(A)*. To enhance the visual detection of the communities, we have represented all singleton communities with the same gray color. *(C)* The consensus partition representing the multi-scale community structure for the group-level functional matrix, which is defined as the average connectivity matrix across participants. Here, again, to enhance clarity, we color the singleton communities in the same gray color. *(D)* The average number (out of 600 total nodes) as well as the average size (expressed as the percentage of total nodes) of non-singleton communities calculated across layers, which in our case track *γ* increments. In these analyses, we used a structural resolution parameter *γ* ∈ [0.95, 1.7], an inter-layer *γ* increment of 0.01, and 75 layers.

In single subjects, we observe that the FC graphs display hierarchical organization where communities branch into smaller sub-communities across a range of topological scales (Fig 4B). Although multi-scale communities can be detected reliably in single subjects, the group-level consensus reveals less robust hierarchical organization (Fig 4C). Indeed, at the group level, communities extend over smaller *γ* ranges before branching (note the speckled nature of the community allegiance matrix). This observation indicates that there is relatively low inter-subject similarity of the hierarchical communities observed in FC graphs. In fact, a large number of brain regions (especially subcortical regions) fail to display any significant intra-layer community allegiance over most topological scales (see S7 and S8 Figs).

The hierarchical community organization in the FC graphs can be described by characteristic curves of community number and size as a function of resolution. Consistent with the trends observed in the SC graphs, as the value of the structural resolution parameter increases, we again observe a rapid increase in the number of non-singleton communities (maximum average number of FC communities = 94.44 ± 20.66 std) and an analogous drop in the average community size (Fig 4D). As the value of *γ* increases farther, the number of communities branching into singletons increases, and thus the number of non-singleton communities decreases. Again, the global branching process is highly structured, with a large number of nodes maintaining their allegiance to hierarchical communities over a long range of *γ* before branching. Nevertheless, in comparison to the SC graphs, the FC graphs display this hierarchical community organization over a smaller range of *γ* values (full width at half max = 28.86 ± 7.1, Fig 3D).

### Heterogeneity in the hierarchical community organization of functional and structural brain networks

Next we aim to explicitly characterize similarities and differences in the hierarchical community organization of functional and structural brain networks. We begin by focusing on the notion of community longevity or stability across topological scales, and we estimate the average number of stable communities that each node belongs to across layers. Such a computation depends on first choosing a mathematical definition of what constitutes a stable community. Pragmatically, we choose a parametric definition in which a stable community is defined as a community that exists across more than *x*% of the *γ* range studied. We refer to the *x*% as a *stability threshold*. Intuitively, graphs with pronounced multi-scale hierarchical organization display many stable communities per node across a wide range of stability thresholds, while graphs with weak multi-scale hierarchical organization display very few stable communities per node. In addition to the number of stable communities, it is also of interest to quantify the variance of this number across nodes in the network. Consequently, graphs with a large variance of these values across nodes are characterized by greater topological heterogeneity than graphs with a smaller variance of these values across nodes.

**Fig 5:**
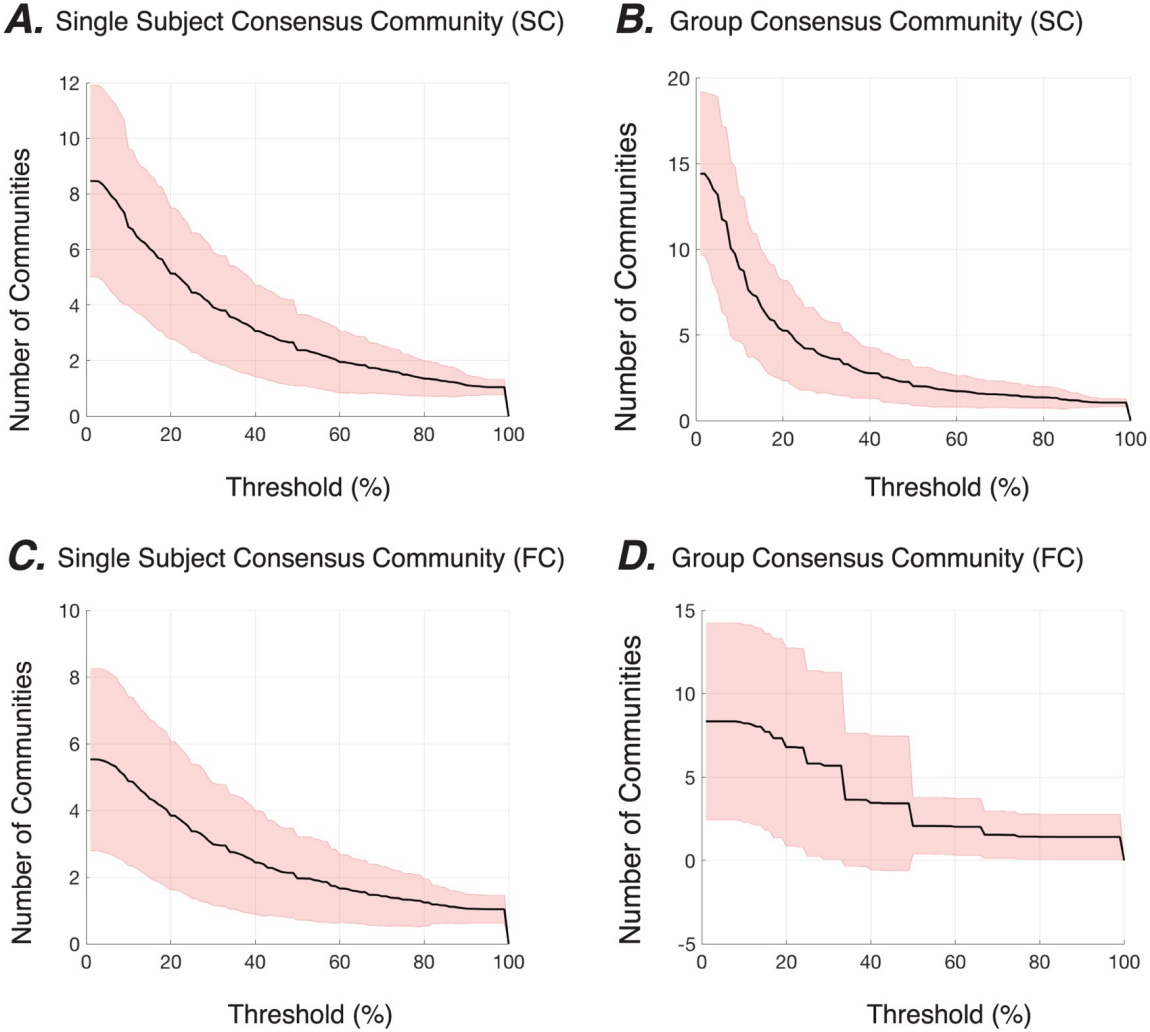
Local topological scales of hierarchical community organization in structural and functional brain graphs. The black lines show the average number of communities to which each node is allied across different stability thresholds in the hierarchical community organization of SC *(A-B)* and FC *(C-D)* graphs, at both the subject level *(A,C)* and the group level *(B,D)*. Shaded areas show standard deviation. We define the stability threshold as the percent range of *γ* values over which a node is stably allied to a given community, here shown along the *x*-axis.

Applying these analyses and statistics to graphs extracted from neuroimaging data, we observe that the brain’s structural and functional connectivity graphs are indeed hierarchical, with the vast majority of nodes displaying stable allegiance to communities over more than one topological scale (Fig 5). First considering only SC graphs, we observe that at low stability thresholds, each brain region is allied to approximately 8 communities across topological scales, while at higher stability thresholds, a brain region may only be allied to 1 community. Across brain regions, we also observe high variance; at low stability thresholds, the number of communities to which a region allies ranges from approximately 5 to approximately 12, indicating a high degree of heterogeneity in the SC graphs. Finally, we observe a marked similarity between the community stability curves at the subject level and at the group level (Fig 5A&5B), providing further evidence of inter-subject similarity of hierarchical community structure in SC graphs.

Next, considering the FC graphs, we observe that brain regions exist in a smaller number of stable communities across layers (Fig 5C&5D). The comparison between the FC and SC subject-level results demonstrate that although the average curves appear similar in shape, the average number of stable communities is approximately 1.5 times higher in SC graphs than in FC graphs. These observations suggest that FC graphs display a flatter hierarchical community organization, characterized by a smaller number of topological scales. Across brain regions, we again observed relatively high variance; at low stability thresholds, the number of communities to which a region allies ranges from approximately 3 to approximately 8, indicating a high degree of heterogeneity in the FC graphs. The community stability curves also display a marked difference at the subject and group levels, again providing evidence of inter-subject variability of hierarchical community structure in FC graphs.

### Reliable detection of a region’s consistent allegiance to communities across topological scales

In the previous sections, we first observed the hierarchical nature of community structure in structural and functional brain graphs, and then we determined the number of topological scales that characterize each node’s community allegiance profiles. In this section, we seek to better understand the fine-scale features of the multi-scale network model that allow for reliable estimation of these topological scales in individual brain regions. Importantly, the multi-scale network model is not simply an agglomeration of weighted adjacency matrices. Rather, it explicitly stitches these matrices together with inter-layer connections that link a brain region in one layer to itself in the preceding and following layers. These inter-layer links allow for the quantitative assessment of communities across layers in a statistically principled manner.

**Fig 6:**
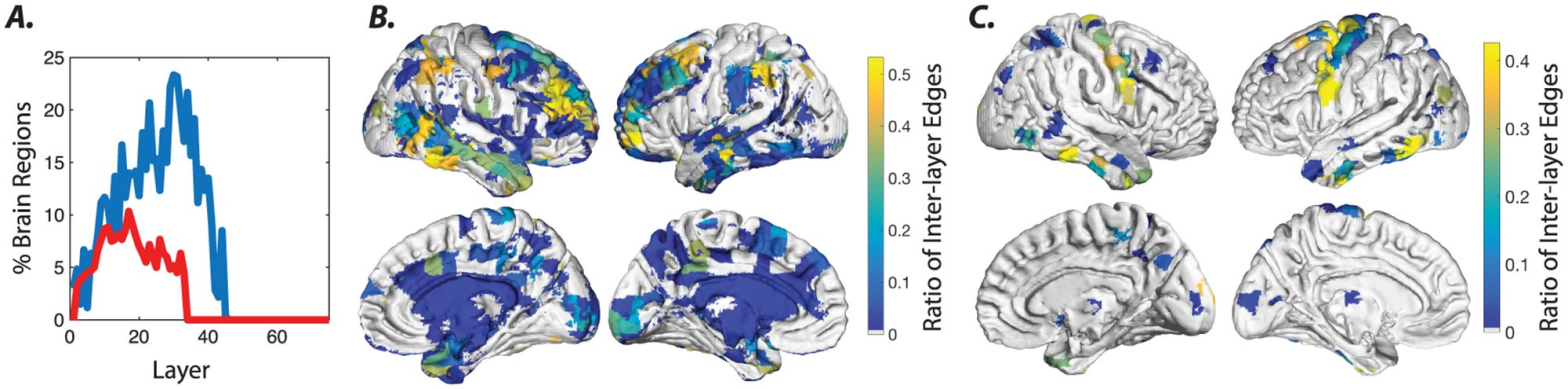
Reliability of inter-layer multi-scale community allegiance of brain regions in SC and FC graphs. *(A)* The percentage of brain regions from the SC (blue) and FC (red) graphs containing inter-layer edges with significant probability at the group-level across layers, which in our case track *γ* increments. The probability of identifying the SC inter-layer edges falls below chance for the second half of the *γ* range values. These results suggest that the estimated stability and the hierarchical structure of the higher *γ* layers are unreliable. Inter-layer coupling weights were (*τ* = 0.50). The probability of identifying the FC inter-layer edges was above the significance level for a small range of *γ* values. Since the vast majority of the group-level consensus inter-layer edges are not significant, the identified communities frequently switch across layers (as seen in (Fig 4C)). *(B-C)* The regions from the SC (panel *(B)*) and FC (panel *(C)*) graphs containing inter-layer edges with significant probability at the group-level are overlayed on the brain and color-coded to represent the ratio of the significant inter-layer edges (i.e., by dividing by the total number of inter-layer edges).

The fact that the multi-scale model contains inter-layer links allows us to assess the inter-layer consistency of a node’s allegiance to communities. In particular, over the large number of optimizations of the modularity quality function that must be performed to adequately sample the underlying landscape, it is of interest to quantify how frequently a node remains in the same community across two adjacent layers (here representative of topological scales). We define a reliable inter-layer association as occurring when a node remains in the same community across two adjacent layers for a greater number of optimizations than expected by chance (see Methods). We observe that a large number of reliable inter-layer associations can be identified in structural brain graphs at both the subject and group levels. This is true particularly for lower values of *γ*, indicating the presence of a *γ* range over which hierarchical community assignments can be reliably detected (Fig 6A). While a number of cortical and most of the subcortical structures including the diencephalon and limbic system display reliable inter-layer association across a small range of *γ* increments at the group-level, several bilateral clusters in the frontal, parietal, and temporal cortex display a notably higher range (Fig 6B). In FC graphs, we observe reliable inter-layer associations over a much smaller range of *γ* at the subject level (Fig 6A). At the group level, we not only observe that reliable inter-layer associations occur over a small range of *γ*, but also that there are very few reliable associations at all (Fig 6C). Together, these findings underscore both the flatter hierarchical nature of FC graphs and the greater inter-subject variability in comparison to SC graphs. Yet, the identified regions from the SC(FC) graphs with reliable inter-layer associations is a reflection of the fact that networks of structures with similar and/or strong structural (functional) connectivity are present across subjects.

### Homogeneity *versus* heterogeneity of topological scales in structural and functional brain graphs

The previous results indicate that we can reliably detect hierarchical community structure in structural and functional brain graphs, and that the two types of graphs display differing degrees of topological heterogeneity. To better understand this heterogeneity, particularly across nodes in the network, we examine the stability of communities more closely. Specifically, for node *i*, we measure the stability of its allegiance to community *‘X’* by calculating the fraction of layers in which node *i* belongs to community *‘X’* (see Methods). This formulation allows us to define a stability matrix by replacing the community label with the stability of the node’s allegiance to the community (S3A and S3B Fig). This matrix quantifies the stability of a node at a given structural resolution parameter value. In this matrix, highly diverse patterns of community allegiance stability are indicative of topological heterogeneity in the graph. By contrast, less diverse patterns of community allegiance stability are indicative of topological homogeneity in the graph.

To quantity homogeneity *versus* heterogeneity, we decomposed the stability matrix using a principal component analysis, such that each component indicated a coherent pattern of communities across scales (S3C and S3D Fig). Intuitively, graphs with greater topological heterogeneity require a larger number of principal components to explain a given amount of variance in the community stability matrix compared to more homogeneous graphs. In both SC and FC graphs, we observed that a handful of components explained most of the variance in the community stability matrix (S3E and S3F Fig). In SC graphs, eight principal components explain more than 95% of the variance in the stability matrix. The first component is marked by the stability profile of the singleton nodes, and the second component highlights the lower half of the *γ* range where most of the larger communities reside. In FC graphs, only five principal components explain more than 95% of the variance in the stability matrix. The group-level analysis shows that, on average, a significantly (*t*-test, *p* < 0.001) smaller number of principal components (4.86 ± 0.78) explain subjects’ FC stability matrices compared to subjects’ SC stability matrices (9.49 ± 1.26). These results provide converging evidence that SC graphs display a greater topological heterogeneity in hierarchical community structure than FC graphs.

### Comparison between the hierarchical community organization of the brain’s structural and functional connectivity

In previous sections, we demonstrate that the hierarchical organization of the SC and FC graphs differ in terms of the presence and stability of communities across topological scales. Yet it is also important to note that the identified communities across modalities do share some similarities, perhaps supporting the notion that structure provides the scaffold for emergent functional dynamics. To better understand the similarities between the hierarchical community organization of SC and FC graphs, we begin by summarizing the community structure at each *γ* value in each modality as an *N* × *N* allegiance matrix, where the *ij*^*th*^ element indicates the fraction of times that node *i* and node *j* are placed in the same community over all optimizations of the multilayer modularity quality function.

**Fig 7:**
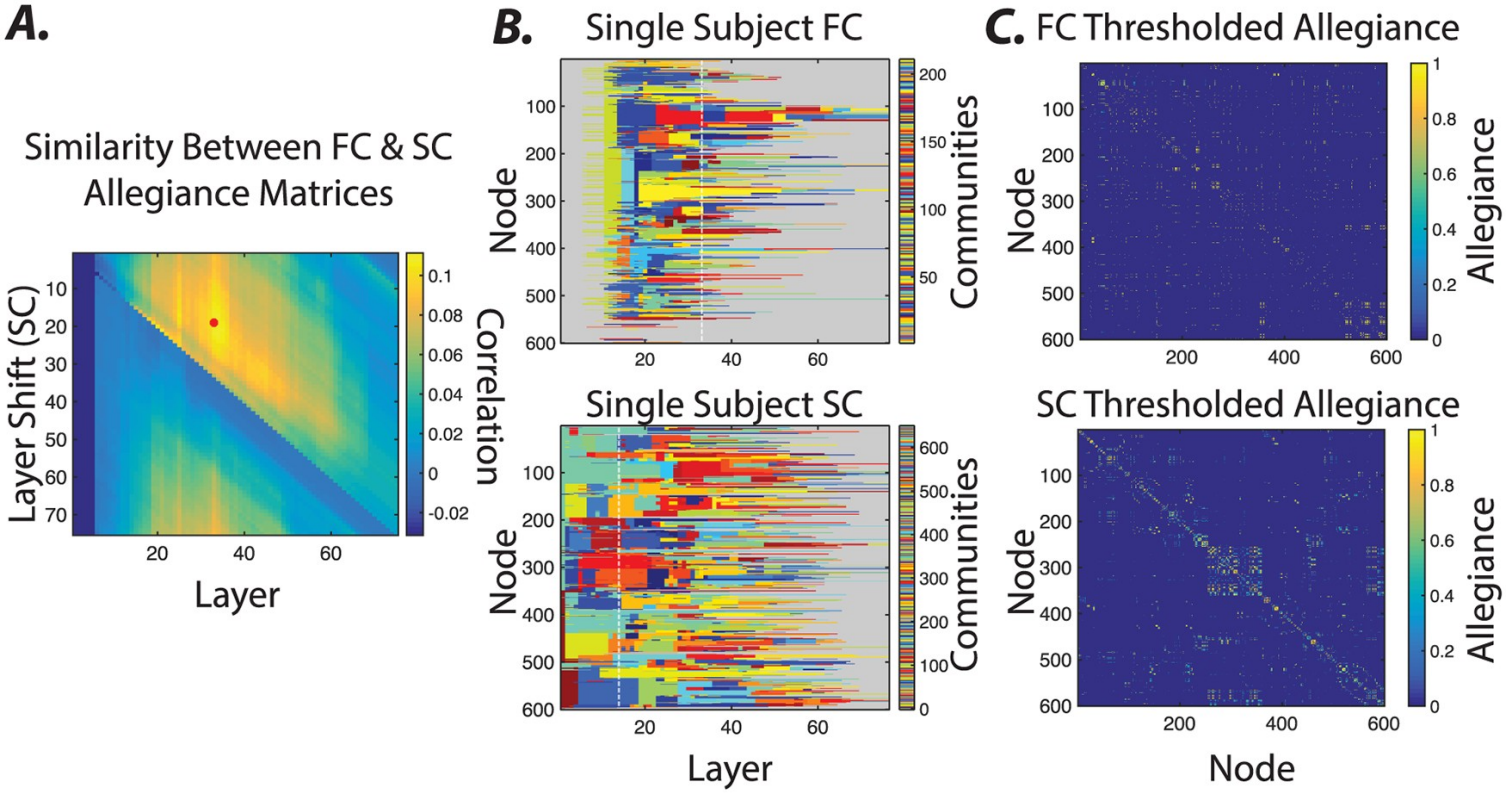
Similarity between the hierarchical community structure of structural and functional brain graphs. *(A)* The similarity between the allegiance matrices of the hierarchical community structures of the FC and SC graphs. Specifically, we calculate the Pearson correlation coefficient between allegiance matrix elements of SC at a given *γ* and the allegiance matrix elements of FC at a given *γ*, for all possible *γ* pairs, allowing us to identify the alignment that results in the highest similarity between their allegiance matrices (marked by * in the plot). Note that a direct comparison between FC and SC allegiance matrices across corresponding layers shows relatively small correlation values (first row). Nevertheless, shifting the SC matrix towards higher layers yields higher correlation values and thus a better allegiance between FC and SC matrixes across topological scales. The dashed white lines in panel *(B)* highlight the layers where the SC and FC realignment yield highest similarity values. *(C)* The thresholded allegiance matrices of layers highlighted by dashed lines in panel *(B)* of the FC and SC hierarchical communities. FC and SC allegiance matrices are ordered identically based on their original node labels.

Next, we calculate the Pearson correlation coefficient between the allegiance matrix of SC at a given *γ* and the allegiance matrix of FC at a given *γ*, for all possible *γ* pairs (Fig 7A). This approach enables us to capture the degree to which densely connected communities of brain regions in FC similarly echo their underlying SC, thereby providing insight into the structural drivers of global dynamics. We observe that SC and FC communities show highest similarity at medium topological scales (Fig 7B), suggesting that it is not simply the case that densely structurally connected regions are also functionally connected. Instead, these results suggest that medium-sized bundles that link the densely connected (and commonly local) brain regions allow global functional synchronization between relatively large ensembles (Fig 7C).

### Explicit multimodal investigation using a multiplex, multi-scale graph

Comparing networks is an important area of methodological development in neuroimaging [64, 65]. While the comparisons thus far between structural and functional brain graphs have been illuminating, it is natural to ask whether there is a more principled and model-based approach to comparing the two modalities within the multilayer framework. Indeed, the multilayer framework does allow additional graphs to be interconnected along distinct dimensions. Thus, it is possible to construct a multiplex graph; in one dimension of this graph, the layers could correspond to topological scales, while in another dimension of this graph, the layers could correspond to the type of connectivity (e.g., structural or functional).

Here we construct exactly this multiplex graph to more formally study the multi-scale nature of both the structural and functional connectivity matrices within the same model (S9A Fig). In addition to inter-scale links *τ*, this model also contained inter-modality links *κ* that link a node in one scale and one modality to itself in the same scale in a different modality. We optimize the modularity quality function in this multiplex case to identify the hierarchical community structure of the SC and FC graphs. Importantly, as *κ* is tuned down, community structure is allowed to be independent in the SC and FC graphs (S9B Fig). In contrast, when *κ* is tuned up, community structure is forced to be consistent across the two types of graphs (S9F Fig). In other words, by employing higher values of *κ*, we are able to extract community structure that is most representative of the graphs in both imaging modalities.

Interestingly, we observe that this cross-modality community structure appears more similar to the community structure of the SC graphs when they were studied independently, than to the community structure of the FC graphs when they were studied independently. This phenotype can occur in community detection when the community structure in one graph is stronger than the community structure in the other graph. To investigate and more thoroughly quantify this observation, we study the similarity between the allegiance matrices of the multiplex SC-FC graph, the FC graph alone, and the SC graph alone, as a function of the topological scale (*γ* value) at which they were constructed (S10 Fig). We observe that the hierarchical structure of the multiplex SC-FC graph is similar to that of the FC graph alone only in a narrow range of topological scales, consistently across *κ* values (S10A and S10B Fig). In contrast, we observe that the hierarchical structure of the multiplex SC-FC graph is similar to that of the SC graph alone across a wide range of topological scales, and consistently across *κ* values (S10C and S10D Fig). These results suggest that the joint optimization is more heavily influenced by the hierarchical community structure in the SC graph than it is by that of the FC graph.

**Fig 8:**
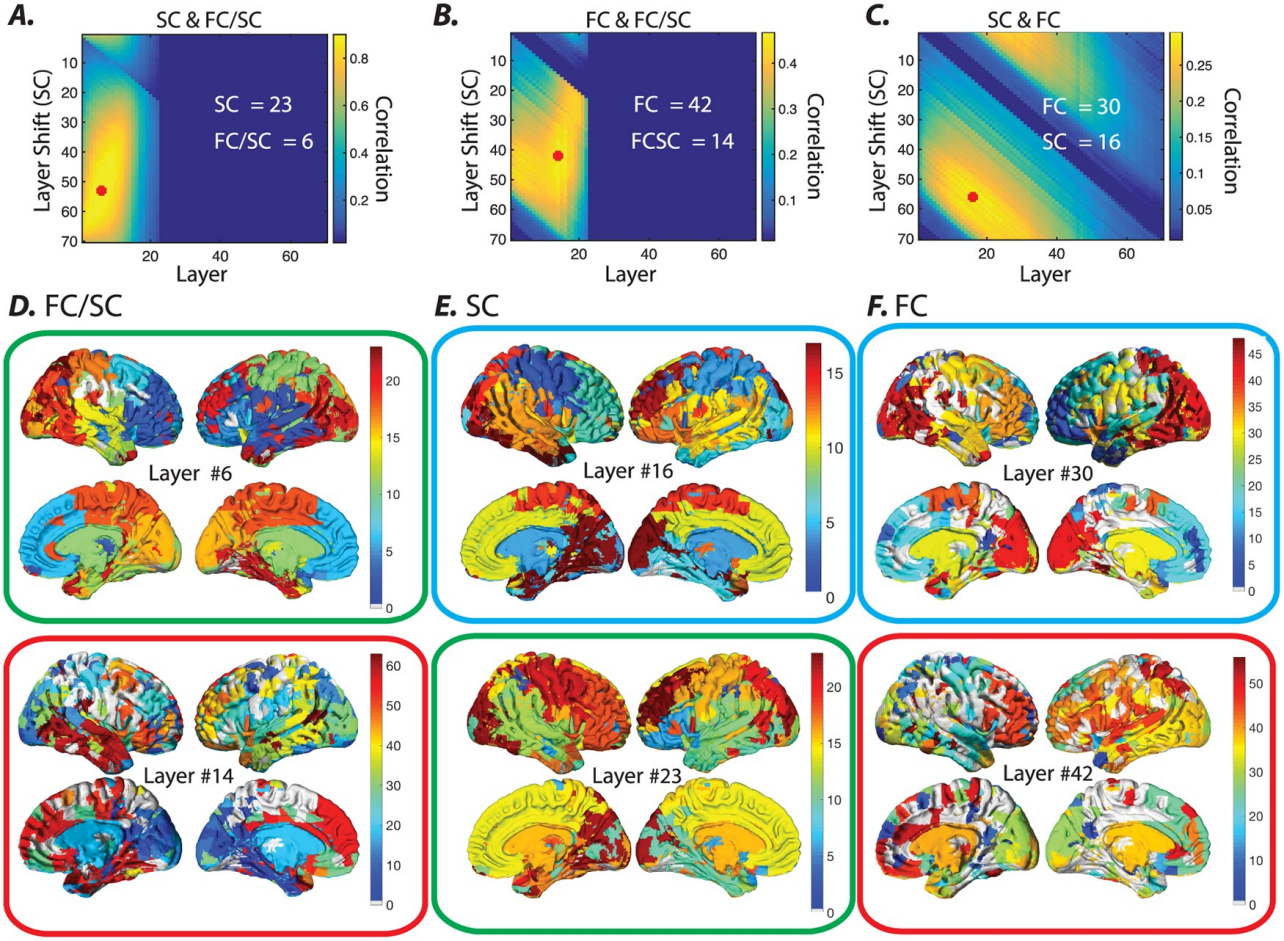
Similarity between the allegiance matrices of the multiplex SC-FC graph, the FC graph alone, and the SC graph alone, as a function of the topological scale for the group. Using a (*κ* = 1), we calculated the Pearson correlation coefficient for all layers and all layer shifts between the allegiance matrices of *(A)* the multiplex SC-FC graph and the SC graph, *(B)* the multiplex SC-FC graph and the FC graph, and *(C)* the SC graph and the FC graph outside of the multiplex formulation. Layers and shifts with maximum correlation values are marked by *. The layer numbers that yield maximum correlation values are also presented on the plots (white). *(D-F)* Brain overlays show the communities identified at layers with maximal correlation values (panels *(A-C)*). The color bars in each panel (right hand side) represent the color-coded communities. The multiplex SC-FC communities at low *γ* layers are very similar to SC communities at slightly higher *γ* layers (green boxes). Although FC communities overall show smaller similarity to the muliplex SC-FC communities, the multiplex SC-FC communities at higher *γ* layers show relatively higher similarity to FC communities at high *γ* layers (red boxes). The smallest similarity is observed between FC and SC graphs, peaking around a coarse scale with small *γ* values for both modalities (cyan boxes).

Importantly, these results are reported over a single subject, and thus it is critical to ask to what degree these insights hold over the entire participant cohort. To address this question, we perform the same set of analyses, but instead of using the single-subject allegiance matrices, we use the group-level allegiance matrices. In general we observe consistent results at the group scale. Specifically, the hierarchical community structure of the muliplex SC-FC graphs at high *κ* values are consistently reminiscent of the SC structure (highest observed correlation approximately *r* = 0.85), along a range of topological scales (Fig 8A). And they are reminiscent of the FC structure to a weaker degree (highest observed correlation approximately *r* = 0.41), along a much narrower range of topological scales (Fig 8B). Notably, the similarity between either SC or FC and the multiplex SC-FC graphs is higher than between SC and FC alone (highest observed correlation is approximately *r* = 0.25). The FC and SC communities at coarse topological scales show the highest similarity (Fig 8C–8F). However they diverge at higher topological scales as the SC communities branch into smaller local communities (S5 Fig), whereas the FC communities at higher topological scales are more spatially distributed. These results confirm at the group level that the joint optimization is more heavily influenced by the hierarchical community structure in the SC graph than it is by that of the FC graph. In addition, regions that display comparable community allegiance between FC and SC graphs such as the subcortical nodes and some clusters within the medial frontal and medial occipital cortices are also identifiable in the multiplex SC-FC graph’s multi-scale communities as they maintain their community allegiance across *γ* increments. Overall the observed similarity between the multiplex SC-FC graph’s communities and the FC and SC communities provides converging evidence that both modalities share major organizational features. Nevertheless the multiplex SC-FC graph’s communities are comparable to the original FC and SC communities at different hierarchical scales, which highlights the differences in the hierarchical community organization of the SC and FC graphs.

## Discussion

The human brain is a complex system that can be fruitfully represented as a graph or network in which brain regions correspond to network nodes and structural or functional connections between regions correspond to network edges [20, 66]. Recent observations have pointed to the fact that both structural and functional brain networks may have community structure [67]: the presence of densely interconnected groups of regions that might support specific cognitive functions [68–70]. Moreover, evidence suggests that these communities exist over multiple topological scales [1], with larger communities potentially being composed of smaller communities [19]. Yet a comprehensive characterization of this putative hierarchical community structure in structural and functional brain graphs has remained difficult largely due to inadequacies in existing analytical paradigms and computational tools. Here we address these limitations by exercising a multi-scale community detection algorithm [31], and applying it to structural brain networks estimated from diffusion imaging and to functional brain networks estimated from resting state fMRI. Using novel statistics including community stability and inter-scale reliability, we show that structural brain graphs display a wider range of topological scales than functional graphs. We also illustrate the utility of this method in examining multimodal graphs that combine both structural and functional connectivity information. Our work illustrates the practical utility of multi-scale community detection in revealing hierarchical community structure in brain graphs, and opens the door for future investigations of this structure in cognition, development, aging, and disease.

### Detecting multi-scale community structure

Characterization of multivariate dependencies across spatio-temporal scales is critical for a fundamental understanding of observable dynamics across systems as diverse as the climate system [71] and the human brain [19]. The multi-scale community detection algorithm that we exercise here reveals the hierarchical community organization of a graph by assuming dependence between neighboring topological scales [31]. A marked advantage of this approach compared to conventional single-scale algorithms is that it directly addresses the question: “Is a community at one scale the same as or different from a community at another scale?” Perhaps even more importantly, the approach provides an estimate of the stability of local topological structure, and therefore a pragmatic means of identifying model parameter values that maximize the consistency of locally stable communities across several topological scales. These local estimates of community stability (unlike the global measures of community stability that have been previously defined [32, 72–75]) are robust to topological heterogeneities in the form of communities of different sizes with different average edge weights [76]. When applied to human brain networks, we find that the local community stability estimates allow characterization of communities that are stable across a range of topological scales. Taken together, our study offers not only a methodological approach to studying hierarchical community structure in graphs, but also a set of statistical methods to characterize the observed structure and to compare it across different classes of graphs, either treated separately or combined into a multiplex model.

### Multi-scale community structure in the human brain’s white matter architecture

Structural brain graphs estimated from diffusion imaging data tend to be sparse, and the edge weight distributions tend to be heavy-tailed [14, 51, 77]. These characteristics can occur when a complex topology is embedded into a 3-dimensional space [1], in such a way as to enhance the efficiency of information transmission [78] while decreasing the cost of wiring [79]. Interestingly, prior work has also offered initial evidence that the complexity of structural connectivity is in part due to the fact that it is organized in a hierarchically modular fashion [14], which is thought to support its information processing capabilities [2]. Here we use a principled mathematical modeling approach to more exactly identify hierarchical community structure in structural brain graphs. Our results demonstrate that structural connectivity is characterized by heterogeneous multi-scale communities, and by nodes that form stable hierarchical communities across a range of topological scales. Multi-scale communities appear to be largely consistent across different subjects and also tend to be spatially localized. The observation of both regional heterogeneity and diverse topological scales indicates that the application of single-scale community detection techniques is likely to produce an overly-simplified picture of the brain’s organization.

While the majority of multi-scale communities were spatially localized, communities in basal ganglia-thalamo-cortical circuitry were not. Over several topological scales, subcortical structures including basal ganglia (mainly putamen, palladum, and caudate) and anterior thalamus as well as several frontal neocortical areas were identified within the same community. While frontal and subcortical structures are spatially distributed, it is commonly known that much of the cortex (including both allocortex and isocortex) projects to the striatum [80], although not all projections are entirely reciprocal. These consistent projections can manifest as structural motifs that are accessible to community detection algorithms. Other complementary algorithms based on tools from algebraic topology, including the notion of persistent homology [81], have demonstrated that basal ganglia-thalamo-cortical connections are among the few most common *cycles* identifiable in structural brain graphs [82]. These cycles and motifs are known to play key roles in rhythmic gain control, and in the gating and integration of information across the brain [83, 84]. For example, basal ganglia influence cortical states and behavior via dopaminergic inputs to thalamus, thereby enabling the integration of information characteristic of reinforcement learning [85, 86]. Indeed, basal ganglia input is modulatory and also serves to gate higher-order relay signals that are propagated through cortico-thalamo-cortical loops [87]. Together, the unique role that the basal ganglia-thalamo-cortical pathways serve in driving brain states is made possible through the unique structural fingerprint of subcortical regions.

### Multi-scale community structure in the human brain’s resting state functional connectivity

The pattern of phase-locking between regional BOLD time-series over a period of several minutes demonstrates that many regions display high functional connectivity with one another [88]. While the resulting graph is relatively dense and homogenous, it nevertheless displays some amount of hierarchical community structure. In single subjects, the hierarchical consensus analysis revealed a small range where multi-scale community structure is reliably identified. Interestingly, the group-level consensus analysis showed that the majority of brain regions failed to produce reliable inter-layer links across individuals, indicating the high degree of inter-subject variance in hierarchical community organization. These results are not entirely unexpected in light of the mounting evidence for both inter-session and inter-subject variability in resting state functional connectivity [89], particularly that located in heteromodal association areas [90, 91]. Speculatively, it is possible that some of this inter-subject variability is due to the fact that these heteromodal association areas are more susceptible to and likely influenced by environmental factors, a fact highlighted by research on the postnatal period where they display protracted development during a time period of high plasticity [90, 92, 93]. Thus, the observed inter-subject variability in the multi-scale community structure of functional brain graphs could provide important fodder for a fundamental understanding of the principles of brain wiring, evolution, and ontogenetic development [93].

### A comparison of hierarchical community structure in functional and structural graphs

It has long been observed that resting state functional connectivity shows statistically similar patterns to those observed in underlying structural connectivity [51, 94, 95]. Yet, we have little understanding of exactly how the anatomical connections gives rise to observed functional interactions [42]. Evidence suggests that the relationship between structure and function is likely quite indirect [96], with the broader network adjacent to direct structural paths being critical to healthy dynamic couplings between brain regions [97]. Our work supports this notion by demonstrating that the highest similarity in hierarchical community structure between the two modalities is found between the fine topological scales of the functional graph and the relatively coarse topological scales of the structural graph, which takes into account broader anatomical network organization. The differential scales of function and structure that map onto one another can in part be explaind by the observation that the structural graphs that we define in this work display on average ≈ 1.5 times more topological scales than the functional graphs defined in this work. It will be important in future to determine the consistency of this finding across different methods of defining both structural and functional graphs. Nevertheless, more broadly these results suggest that (i) functional connectivity dynamics are not strictly bound to or constrained within direct anatomical projections, but instead extend to spatially distributed circuits, and (ii) structural connections as estimated by white matter tractography display hierarchical community structure that can support long range functional coupling [11, 98].

It is important to note that although we found similarities between the hierarchical community structure of functional and structural graphs at different topological scales, the overall magnitude of the similarity was relatively small. One fundamental property of brain connectivity that might explain this relative independence of structural and functional graphs is temporal dynamics [45, 49, 99]. Indeed, while we have here studied a static functional graph that represents patterns of co-activation over several minutes, in reality the brain displays time-varying patterns of functional connectivity [100] that can track changes in cognitive processes [101] and behavior [102, 103]. The nature of these dynamics suggest that the answer to the question “How does structure constrain function?” depends nontrivially on the time scale of the function (or functional connectivity pattern) in question. Indeed, recent work has demonstrated that synchronization patterns in hierarchical modular structures such as the brain may appear at different topological scales depending on the time scale of their interaction [19, 104–108]. Therefore, comparing the topology of the multi-scale functional and structural graphs can prove useful for understanding the properties of anatomical projections that are critical for the emergence of multi-scale functional patterns.

### Explicit multimodal models of the brain’s hierarchical community structure

While the method that we propose and exercise is applicable to brain graphs constructed from a single imaging modality, it is also flexible and generalizable to questions that require fusion of brain graphs constructed from two or more modalities. We illustrate the method’s utility in this class of problems by exploring the joint optimization of modularity across a multiplex network composed of both functional and structural layers [109]. This construction enables us to highlight the characteristics of community structure that are echoed across the two imaging modalities: diffusion imaging and resting state functional MRI. Our results reveal that at high inter-modal coupling strengths, the community organization of the multi-modal graph merges across modalities to form a hybrid structure. Similarity analysis revealed that the multi-modal graph shows highest similarity to functional graphs at relatively fine topological scales and to structural graphs at relatively broad topological scales. The narrow range of topological scales at which this hybrid structure was identified highlights the fact that community structure in functional dynamics extends beyond direct white matter projections. This work complements prior efforts to bridge functional and structural connectivity patterns using tools and techniques that span the purely qualitative and the exquisitely quantitative: these approaches include direct superposition, fiber tracking from functional parcels, and regression analysis relating functional, anatomical, and behavioral data [110]. One recent study identified communities separately for each modality, and then subsequently maximized a cross-modularity function to identify a community partition shared by structure and function at medium-to-fine topological scales [111]. Our work extends these findings by assessing hierarchical community organization in a multiplex network composed of both functional and structural layers. Future work could seek a better understanding of how joint optimization of graphs with varying topologies across modalities could lead to the robust detection of shared features.

### Methodological considerations

Several methodological considerations are pertinent to this work. First, although the multi-scale community detection algorithm identifies the entire hierarchical community structure simultaneously, the resolution at which we study the topology of the graph is limited by the number of layers in the multi-scale graphs. As a natural consequence, high resolution multi-scale community detection can be computationally intensive for very large graphs. This property can be especially limiting in multi-modal graphs where two graphs show widely different hierarchical organizations, thus making it time-intensive to accurately sample the modularity landscape. Second, one of the greatest advantages of using conventional community detection algorithms is that they provide a single partition of nodes into communities. Using this partition we can extract additional information regarding the role that individual nodes play within the graph by calculating summary statistics including the participation coefficient and within-module degree *z*-score [112]. However, there are currently no equivalent summary statistics for multi-scale community organization, and therefore the utility of this approach could be significantly enhanced by the parallel development of such statistics. Third, the modularity quality functions that we studied in this work incorporate a uniform null model [56] but future work could explore different null models for multi-scale and multi-modal communities [113], with the goal of determining their relative utility in extracting hierarchical community structure from brain graphs. Moreover, in an effort to align the hierarchical community organization of the functional matrices across subjects, we used the average edge weight of each matrix as the value for its uniform null. There may, however, be other reasonable choices for variables that fulfill the criterion of displaying low variance across subjects. Future work could fruitfully explore alternative methods to align hierarchical communities across subjects to allow the identification of functionally relevant topological scales. Fourth, it is worth noting that due to computational limitations, we only examined low values of the inter-layer (scale) dependence. Future work could extend our observations by assessing hierarchical community structure apparent at different values of the inter-layer weight, or when linking layers either ordinally or categorically [31]. Fifth, we note that functional graphs and structural graphs are constructed from inherently different imaging data, and involve the choice of different generative parameters and methods. Thus, the comparisons between them that we provide in this work are statistical in nature. Sixth, we note that the community structure identified with the multi-scale community detection algorithm can provide inherently different and complementary information to that provided by single-scale algorithms; as we show in prior work, the fact that the modularity quality function can be altered is useful for testing distinct hypotheses, and different sorts of community structure can be identified with different modularity quality functions [114]. Finally, it will be interesting in future to use this multi-scale community detection algorithm to better understand the development of structural and functional graphs in childhood, their relation to cognitive function, and their alteration in disease.

We also note that other hierarchical community detection methods exist [115–120], and it is important to understand the similarities and differences between our method and other important and useful methods that are currently available in the literature. There is significant work in the literature linking different static (non-multilayer) community detection techniques, including quite elegant work based on Markov processes. Specifically, Delvenne and colleagues define the stability of a partition as a measure of the partition’s quality as a community structure based on the clustered autocovariance of a dynamic Markov process taking place on the network [72]. They show that modularity maximization can be understood in relation to the stability at *t* = 1, while spectral clustering based on the normalized Fiedler vector can be understood in relation to the stability as *t* → ∞. Furthermore, Markov time can act as a resolution parameter similar to the structural resolution parameter in modularity maximization. While the two approaches therefore have some theoretical connection, there are some pragmatic differences that could motivate the use of one over the other. Here we raise 3 such differences, but note that a direct comparison of our method to those based on Markov processes in these same data would be an interesting focus for future work. First, many other existing methods require an additional algorithm to link communities across scales; such algorithms can vary from simplistic ones based on community similarity to ones that employ more sophisticated approaches. In contrast, our method automatically incorporates information across scales to inform the identified multi-scale community structure, and the strength of this incorporation can be tuned via the *τ* parameter. Second, the multi-scale community detection method that we exercise here provides a particularly interpretable community structure of human brain networks, where the topological scales have a direct relationship to the edge weights (which have a specific neurobiological meaning), thereby accurately delineating local communities at their relevant topological scale. Third and finally, the muti-scale community detection method allows for an accurate characterization of the stability of local communities, which nicely complements the Markov time approach that instead provides a characterization of the global stability of all communities.

## Conclusion

In this work, we examined a multi-scale community detection algorithm and its advantages for uncovering the hierarchical organization of synthetic and real world graphs. By assuming dependence between the adjacent topological scales, the multi-scale algorithm links the communities persisting over several scales, thereby effectively uncovering hierarchical community organization in graphs. We demonstrated the statistical robustness of this hierarchical organization by defining notions of community stability and inter-scale reliability. After exercising the method on synthetic graphs, we next examined and compared the hierarchical community organization of structural brain graphs and functional brain graphs estimated from diffusion imaging and resting state functional MRI, respectively. Compared to the functional graphs, the structural graphs displayed a higher degree of topological heterogeneity with a more pronounced hierarchical organization as evidenced by a higher average number of stable communities across topological scales. With the exception of basal ganglia-thalamo-cortical circuitry, the structural communities across topological scales tended to be spatially localized, where nodes within a community were located in close physical proximity to one another. Interestingly, functional communities displayed weak similarity to structural communities at coarse topological scales, and this dissimilarity became more pronounced at finer topological scales as spatially distributed functional communities emerged. These statistical differences between the spatially distributed functional communities and spatially localized structural communities were also apparent in an explicit multi-modal extension of our method, which performs a joint optimization of modularity across a multiplex network composed of both functional and structural layers. Taken together, these results illustrate the practical utility of multi-scale community detection in revealing hierarchical community structure in single-modality and multi-modality brain graphs.

## Appendix

## 1 Spectral community detection of dynamic (multi-slice) networks

The single layer modularity quality function has been generalized to multi-slice networks to identify communities in multiplex or time-dependent networks. Formally, the multi-slice modularity quality function can be defined as

$$\begin{array}{c} Q=\frac{1}{2\mu }\sum_{ijlr}\{(A_{ijl}-\gamma_{l}P_{ijl})\delta_{lr}+\delta_{ij}\omega_{jlr}\}\delta (g_{il},g_{jr}), \end{array}$$ (3)

where the adjacency matrix of layer *l* has components *A*_*ijl*_, the element *P*_*ijl*_ gives the components of the corresponding layer-*l* matrix for the null model, *γ*_*l*_ is the structural resolution parameter of layer *l*, *g*_*il*_ gives the community assignment of node *i* in layer *l*, *g*_*jr*_ gives the community assignment of node *j* in layer *r*, *ω*_*jlr*_ gives the connection strength (i.e., an *inter-layer coupling* parameter) from node *j* in layer *r* to node *j* in layer *l*, the total edge weight in the network is $\mu =\frac{1}{2}\sum_{jr}K_{jr}$, the strength (i.e., weighted degree) of node *j* in layer *l* is *K*_*jl*_ = *k*_*jl*_ + *C*_*jl*_, the intra-layer strength of node *j* in layer *l* is *k*_*jl*_ = ∑_*i*_ *A*_*ijl*_, and the inter-layer strength of node *j* in layer *l* is *k*_*jl*_ = ∑_*r*_ *ω*_*jlr*_.

Here we extend the multi-scale framework to multi-slice networks. Formally the multi-slice multi-scale modularity quality function we study can be defined as

$$\begin{array}{c} Q=\frac{1}{2\mu }\sum_{ijlrxy}\{(A_{ijl}-\gamma_{l}P_{ijl})\delta_{\left(lx,ry\right)}+\delta_{\left(ix,jy\right)}\omega_{jlrx}+\delta_{\left(il,jr\right)}\tau_{jxy}\}\delta (g_{ilx},g_{jry}), \end{array}$$ (4)

where the adjacency matrix of layer *l* has components *A*_*ijl*_, the element *P*_*ijl*_ gives the components of the corresponding layer-*l* matrix for the null model, *γ*_*l*_ is the structural resolution parameter of layer *l*, *g*_*il*_ gives the community assignment of node *i* in layer *l*, *g*_*jr*_ gives the community assignment of node *j* in layer *r*, *ω*_*jlrx*_ gives the connection strength from node *j* in layer *r* to node *j* in layer *l* at scale layer *x*, *τ*_*jxy*_ gives the *topological scale coupling parameter* which indicates the strength of the links between neighboring topological scales (as represented by layers), from node *j* in scale layer *x* to node *j* in scale layer *y*, the total edge weight in the network is $\mu =\frac{1}{2}\sum_{jrx}K_{jrx}$, the strength (i.e., weighted degree) of node *j* in layer *l* and scale layer *y* is *K*_*jly*_ = *k*_*jly*_ + *C*_*jly*_+ *T*_*jly*_, the intra-layer strength of node *j* in layer *l* and scale layer *y* is *k*_*jly*_ = ∑_*i*_ *A*_*ijl*_, the inter-layer strength of node *j* in layer *l* and scale layer *y* is *C*_*jly*_ = ∑_*r*_ *ω*_*jlry*_, and the inter-scale strength of node *j* in layer *l* and scale layer *y* is *T*_*jly*_ = ∑_*x*_ *τ*_*jlxy*_.

Finally we provide a synthetic example in S11 Fig to show how the multi-scale community detection algorithm links communities across temporal scales and to uncover the relationships between them.

## 2 Hierarchical community organization of synthetic graphs

Here we provide synthetic examples of graphs where each node can be identified locally within a community at three different topological scales. Next we create variations in the hierarchical organization of the graphs by systematically introducing edge strength inhomogeneities. As seen in S4 Fig, multi-scale communities and the relative stability of communities across scales clearly uncovers the planted relationships and inhomogeneity profile across nodes.

## 3 Principal components analysis of the SC and FC stability matrices

Here we use principal components analysis (PCA) to assess the stability profiles of nodes across *γ* increments (i.e. layers) and measure the topological heterogeneity of the graphs. We used the number of components that account for more than 95% of the variance in the stability matrices as a proxy for topological heterogeneity. Low numbers indicate that most nodes display similar stability profiles and therefore the graph is relatively topologically homogeneous. Conversely, higher numbers indicate that most nodes display diverse stability profiles and therefore the graph is relatively topologically heterogeneous (S3 Fig).

## 4 Hierarchical community organization of a multiplex SC and FC graph

The FC and SC communities share similar community organization, and joint-optimization of FC and SC graphs (i.e. SC-FC multiplex graphs) can in theory be used to evaluate these similarities. That said, the community organization of the SC-FC multiplex graph is highly dependent on the inter-modality coupling parameter, *κ*. In S9 Fig, we demonstrate that at smaller *κ* values the FC-SC graph yields two separate community structures for FC and SC components of the graph. However, for higher *κ* values they both share the same hybrid hierarchical community structure. The direct comparison between the community allegiance matrices of the FC, SC, and SC-FC graphs provided in S10 Fig shows the effect of increasing inter-modality coupling parameter on the SC-FC community structure.

## 5 Sorting nodes based on multi-scale community allegiance

Here we provide a brief note on visualization. Sorting the nodes of the adjacency matrices based on their community allegiance allows us to visualize communities of densely connected nodes. Nevertheless for the multi-scale communities, the order of the nodes can change depending on the topological scale. In an effort to by-pass this limitation and enhance the visualization of these communities we sort the nodes based on the similarity of their community assignments across scales. Specifically, we perform optimal leaf ordering (optimalleaforder.m) for hierarchical clustering (linkage.m) using the distances (pdist.m) calculated between the community assignments of each pair of nodes. All multi-scale community plots in this manuscript were generated using this node sorting algorithm.

## 6 Multi-scale group consensus communities in structural and functional brain graphs

Here we provide the group consensus multi-scale community results for the structural (S5 Fig) and functional (S7 Fig) graphs. One salient feature of the group consensus SC multi-scale community is that the communities are overwhelmingly made up of neighboring brain structures across the entire range of topological scales. To highlight the spatial proximity of the communities of the structural connectivity graphs, we identified (bwconncomp.m) and only presented the communities with more than one cluster in S6 Fig while removing all the other communities with only one cluster of brain regions. Next, we tested the statistical significance of these observations via permutation test (*N* = 1000) across *γ* increments (S12 Fig). Our results demonstrate that the number of SC communities with more than one cluster is significantly (*p* < 0.01, Bonferroni corrected for multiple comparisons) smaller than that of the null distribution (generated by changing the assignment of nodes to communities uniformly at random) for all increments of *γ* (except layer #3). Unlike structural graphs, functional graphs fail to yield group level consensus results for a large number of brain regions, including several subcortical and cortical structures (S7 Fig). We highlight these structures separately in S8 Fig.

## 7 Distribution of edge weights in the structural and functional brain graphs

The distribution of the edges in the structural and functional connectivity matrices are notably different. While the distribution of SC edges are extremely heavy-tailed, the FC edges are close to a Gaussian distribution (S2 Fig).

## Supporting information

S1 Fig Effect of the topological scale coupling parameter on the identified hierarchical community structure. ***(A-F)*** Panels show the multi-scale communities of the synthetic graph in Fig 2 of the main text, detected when using different values of the topological scale coupling parameter (*τ*). Here we similarly used a *γ* ∈ [0, 12], an inter-layer *γ* increment of 0.05, and 241 layers. Intuitively, as the value of *τ* tends toward 0, the method identifies community structure that is independent across topological scales, while at high values of *τ* the method identifies a community structure that is largely shared across topological scales (here observable at *τ* = 100). Clear hierarchical structure, where small modules are nested inside larger modules, is accessible at middling values of *τ*, here spanning the range from 0.001 to 10.
(EPS)

S2 Fig Distribution of edge weights in the structural and functional brain graphs. The distribution of streamline counts for all edges in the structural brain graphs at the subject level *(A)* and at the group level *(B)*. Note the heavy-tailed distribution of streamline counts where hundreds of edges are made up of only a few streamlines while only a handful of edges are made up of hundreds of streamlines. Conversely, the functional brain graphs both at the subject level *(C)* and at the group level *(D)*) display a sharp Gaussian distribution where the vast majority of edges share similar values around the mean.
(EPS)

S3 Fig Principal components analysis of the SC and FC stability matrices extracted from a single representative subject. *(A)* The SC stability matrix and *(B)* FC stability matrix created by exchanging the community labels with their corresponding stability values. *(C&D)* Principal components analysis of the matrices shown in panels *(A&B)*, with eight principal components shown for the SC stability matrix and five principal components shown for the FC stability matrix. *(E&F)* We observe that only a small number of principal components account for most of the variance: eight components in the SC stability matrix and five components in the FC stability matrix account for 95% of the variance in the SC and FC stability matrices. Together, these results demonstrate that SC graphs are topologically more heterogeneous than FC graphs.
(EPS)

S4 Fig Synthetic graphs with diverse hierarchical structure. The adjacency matrices (*Left*) and their corresponding hierarchical community structure identified using the multi-scale method (*Right*) are presented for three different graphs (A,C,E) where nodes form hierarchical communities at three separate topological scales. In graph (*A*), the communities at coarse (and fine) topological scales are similarly identifiable across all nodes. However, the medium scale communities show differential discriminability as we have introduced node strength heterogeneity by assigning different within-community edge values for each community at that scale. The discriminability of these communities echoes the stability of the community branches: where stability is defined as the number of layers per branch divided by the total number of layers. The graph in panel (*C*) displays the same three-scale topology as the graph in panel (*A*) except that the medium scale communities have relatively higher discriminability, due to higher within-community edge weight at this scale. The higher stability of the branches of the multi-scale community structure (*D*) similarly echoes the observation in panel (*C*)). The example in panel (*E*) is a graph where we introduced node strength heterogeneity in coarse- and medium-scale communities. Although the communities do not align at global scales for all nodes (as a result of the topological heterogeneity), the multi-scale community structure accurately preserves the local stability profiles of branches (*F*), effectively revealing the three local topological scales in the graph.
(EPS)

S5 Fig Group consensus hierarchical community organization of structural graphs. The communities at several *γ* values are color-coded and overlaid separately for each scale. Singleton communities are removed from the visualization for clarity.
(EPS)

S6 Fig Spatially disconnected communities of the group-level hierarchical community structure in structural brain graphs. Only the spatially disconnected communities (i.e., multi-cluster communities) at several *γ* values are color-coded and overlaid separately for each scale (for details, see section 1 in the appendix). Singleton communities are removed. Note that several subcortical structures such as the caudate nucleus (Cau), pallidum (Pall), putamen (Put), and thalamus (Thal) form communities with areas in fronto-temporal cortex.
(EPS)

S7 Fig Group consensus hierarchical community organization of functional graphs. The communities at several *γ* values are color-coded and overlaid separately for each scale. Singleton communities are removed from the visualization for clarity.
(EPS)

S8 Fig Regions lacking group-level consensus in functional connectivity graphs’ hierarchical community structure. Brain regions that fail to show significant group-level allegiance with other regions are identified as singleton communities in the consensus communities. The community allegiance of a pair of nodes is deemed significant if the group-level average allegiance of that pair (constructed from the subject-level consensus partitions) exceeds that of the null distribution constructed via randomizing the community label assignments. All singleton communities are color-coded (blue) and overlaid on the cortical surface separately for different *γ* values. These regions include subcortical structures (e.g., basal ganglia and thalamus) and large sections of posterior cingulate and dorsolateral (pre)frontal cortex. Note that these regions are identified as singletons even at coarse topological scales.
(EPS)

S9 Fig Hierarchical community organization of a multiplex, multi-scale graph that explicitly combines both imaging modalities: Structure and function. *(A)* The multiplex graph representing both imaging modalities is produced by coupling the nodes with the same identity across modalities (i.e., across FC and SC graphs). The strength of the coupling between modalities, *κ*, affects the dependence of the hierarchical community structure on both modalities. As *κ* is tuned down, community structure is allowed to be independent in the SC and FC graphs, while when *κ* is tuned up, community structure is forced to be more consistent across the two types of graphs. Panels *(B-F)* demonstrate how increasing *κ* causes a shift from two different hierarchical community structures for the FC and SC components of the multiplex graph *(B)* to a single hybrid hierarchical community structure shared by both FC and SC components of the multiplex graph *(F)*.
(EPS)

S10 Fig Similarity between the allegiance matrices of the multiplex SC-FC graph, the FC graph alone, and the SC graph alone, as a function of the topological scale for a representative subject. *(A-B)* Pearson correlation coefficient values for all layers and all layer shifts between the allegiance matrices of the multiplex SC-FC graph and the allegiance matrices of the FC graph alone, for low (*κ* = 0.2) and high (*κ* = 1) coupling values, respectively. *(C-D)* Pearson correlation coefficient values for all layers and all layer shifts between the allegiance matrices of the multiplex SC-FC graph and the allegiance matrices of the SC graph alone, for low (*κ* = 0.2) and high (*κ* = 1) coupling values, respectively.
(EPS)

S11 Fig Multi-scale community organization of a synthetic dynamic graph. *(A)* We created a dynamic graph by periodically changing the community structure of the graph. In this example, the network starts by switching between two community structures, indicated by the colors red and green. However, at slower time scales the switching dynamics between the red and green community structures periodically changes to an alternative switching dynamics between two different community structures, indicated by blue and orange. In this way, we create dynamics in the graph’s community structure at two different time scales. *(B)* The multi-scale community organization of the synthetic dynamic network. Here we provide the results of the multi-scale dynamic community detection algorithm over several temporal scales. Note that the results at the lower *ω* values uncover fast dynamics whereas the results at the higher *ω* values uncover slow dynamics. *(C)* The community allegiance matrices of the multi-scale communities over low, medium, and high *ω* values. Note that at higher temporal scales, the community allegiance matrices are similar to the combination of community allegiance matrices at lower temporal scales.
(EPS)

S12 Fig Number of spatially disconnected communities of the group-level hierarchical community structure in structural brain graphs. The total number of communities and the total number of spatially disconnected communities (i.e., multi-cluster communities) at each *γ* value are marked by *’o’* and ’*’, respectively. The null distributions (*N* = 1000) of the total number of spatially disconnected communities (created by randomizing the community labels) are marked by ’.’ and presented for all *γ* increments (i.e. layers). Note that—except for layer #3—all the empirical values are significantly lower than that of the null distribution. These results suggest that except for a few distributed networks, the SC communities at low *γ* values mainly consist of a large number of neighboring nodes and branch into smaller communities of neighboring nodes.
(EPS)
